# WNT signaling in pre-granulosa cells is required for ovarian folliculogenesis and female fertility

**DOI:** 10.1242/dev.198846

**Published:** 2021-04-29

**Authors:** Okiko Habara, Catriona Y. Logan, Masami Kanai-Azuma, Roeland Nusse, Hinako M. Takase

**Affiliations:** 1 Laboratory for Organismal Patterning, RIKEN Center for Biosystems Dynamics Research, Kobe 650-0047, Japan; 2 Howard Hughes Medical Institute, Department of Developmental Biology and Institute for Stem Cell Biology and Regenerative Medicine, Stanford University School of Medicine, Stanford, CA 94305, USA; 3 Department of Experimental Animal Model for Human Disease, Center for Experimental Animals, Tokyo Medical and Dental University, Tokyo 113-8510, Japan

**Keywords:** Reproduction, WNT signaling, Folliculogenesis, Oogenesis, Granulosa cells, Oocytes, Mouse

## Abstract

In mammalian ovaries, immature oocytes are reserved in primordial follicles until their activation for potential ovulation. Precise control of primordial follicle activation (PFA) is essential for reproduction, but how this is achieved is unclear. Here, we show that canonical wingless-type MMTV integration site family (WNT) signaling is pivotal for pre-granulosa cell (pre-GC) activation during PFA. We identified several WNT ligands expressed in pre-GCs that act in an autocrine manner. Inhibition of WNT secretion from pre-GCs/GCs by conditional knockout (cKO) of the wntless (*Wls*) gene led to female infertility. In *Wls* cKO mice, GC layer thickness was greatly reduced in growing follicles, which resulted in impaired oocyte growth with both an abnormal, sustained nuclear localization of forkhead box O3 (FOXO3) and reduced phosphorylation of ribosomal protein S6 (RPS6). Constitutive stabilization of β-catenin (CTNNB1) in pre-GCs/GCs induced morphological changes of pre-GCs from a squamous into a cuboidal form, though it did not influence oocyte activation. Our results reveal that canonical WNT signaling plays a permissive role in the transition of pre-GCs to GCs, which is an essential step to support oocyte growth.

## Abstract (precis)

**Summary:** Canonical WNT signaling is required for postnatal oocyte growth by supporting the activation of surrounding somatic cells in the mouse ovary.

## INTRODUCTION

In female mammals, including humans, precise control of folliculogenesis is essential for fertility. Oocytes are protected and grow within follicles, which are the fundamental units of the ovary. Dormant oocytes are arrested at the diplotene stage of meiosis I, reserved in primordial follicles, and surrounded by pre-granulosa cells (pre-GCs) (Pepling, 2006; Pepling and Spradling, 2001). Only a small proportion of primordial follicles is activated concurrently, with activation resulting in follicular growth and the serial development of primary, secondary, preantral and antral follicles. Although primordial follicles are able to survive for years to decades, once activated their lifespan is limited to days to months, with their potential fates being either ovulation or atresia (McGee and Hsueh, 2000). In women with primary ovarian insufficiency (POI), the number of follicles rapidly declines and menopause occurs before the age of 40, resulting in severe fertility problems. POI has an estimated prevalence of 1% in women worldwide. The cause of POI remains unknown in most cases, but misregulation of primordial follicle activation (PFA) is regarded as a contributing factor (De Vos et al., 2010; Jankowska, 2017). Therefore, precise control of PFA is required for maintenance of female reproductive ability, as new oocytes are not thought to be generated after birth (Lei and Spradling, 2013).

Given that functional gonadotropin receptors are not present in primordial follicles, PFA is thought to be controlled in a gonadotropin-independent manner (Mason et al., 1986). PFA is characterized morphologically by oocyte growth to a diameter of >20 μm, and proliferation and transition of the squamous pre-GCs into cuboidal/columnar granulosa cells (GCs). These two events occur synchronously, indicating that oocyte outgrowth and morphological changes of pre-GCs into GCs are highly coordinated (Adhikari and Liu, 2009). Several intracellular signaling pathways in oocytes have been implicated in control of their dormancy or growth. The transcription factor forkhead box O3 (FOXO3) and the phosphatase and tensin homolog deleted from chromosome 10 (PTEN) are required for the quiescence of oocytes, whereas the phosphoinositide 3-kinase (PI3K)–AKT–mammalian target of rapamycin (mTOR) pathway contributes to oocyte activation (Adhikari et al., 2009; Castrillon et al., 2003; John et al., 2008; Reddy et al., 2008). In addition, environmental factors such as hypoxia and mechanical stress can influence maintenance of the dormant state of oocytes (Nagamatsu et al., 2019; Shimamoto et al., 2019). In contrast, the mechanism underlying the activation of pre-GCs is less well understood. Differentiation of pre-GCs was found to be disrupted in mice deficient in *Foxl2* or both GATA binding protein 4 (*Gata4*) and *Gata6*, leading to suppression of the transition from squamous pre-GCs to cuboidal GCs, although gene ablation from the early embryonic period might also affect GC lineage identity (Padua et al., 2014; Schmidt et al., 2004). The transition of pre-GCs to GCs is likely to trigger PFA, given that the associated activation of the mTOR pathway in pre-GCs results in the production of Kit ligand (KITL), which contributes to oocyte activation (Liu et al., 2014). However, a comprehensive understanding of the mechanism of PFA requires clarification of how the transition of pre-GCs to GCs is regulated during this process.

Wingless-type MMTV integration site family (WNT) signaling is an evolutionarily conserved system for cell-cell communication, which is classified broadly into canonical [β-catenin (CTNNB1)-dependent, also referred to as WNT/β-catenin signaling] and noncanonical (CTNNB1-independent) pathways (Martin-Orozco et al., 2019). Nineteen WNT ligands have been identified and contribute to diverse processes such as development, stem cell control, and disease in mice and humans (Nusse and Clevers, 2017; Wiese et al., 2018). Whereas WNT4-mediated canonical WNT signaling has been shown to be important for sex determination during embryonic development (Parma et al., 2006; Vainio et al., 1999), the function of WNT signaling during postnatal folliculogenesis remains unclear. Both *Wnt2* knockout and GC-specific *Wnt4* knockout female mice were found to have slightly reduced fertility, with the mild nature of this defect in each case likely being due to functional redundancy among WNT ligands (Boyer et al., 2010; Monkley et al., 1996). More recently, oocyte-derived R-spondin 2 (RSPO2) was shown to contribute to the activation of WNT signaling in GCs (De Cian et al., 2020). RSPO2 is a WNT agonist that is secreted extracellularly and enhances canonical WNT signaling (Kazanskaya et al., 2004), and follicle growth was found to be impaired in ovaries with loss of RSPO2 function in transplant experiments (De Cian et al., 2020). Although WNT signaling is implicated together with other important factors such as GDF9 (growth differentiation factor 9) and BMP15 (bone morphogenetic protein 15) in PFA (Dong et al., 1996; Dube et al., 1998), its mechanism of action has been unknown. Here, we reveal an essential role of canonical WNT signaling in regulation of the transition of pre-GCs to mature GCs during PFA by focusing on postnatal folliculogenesis and taking advantage of mouse mutants that avoid the issue of the redundancy of WNT ligands.

## RESULTS

### Canonical WNT signaling in pre-GCs is essential for female fertility

**Fig. 1.**
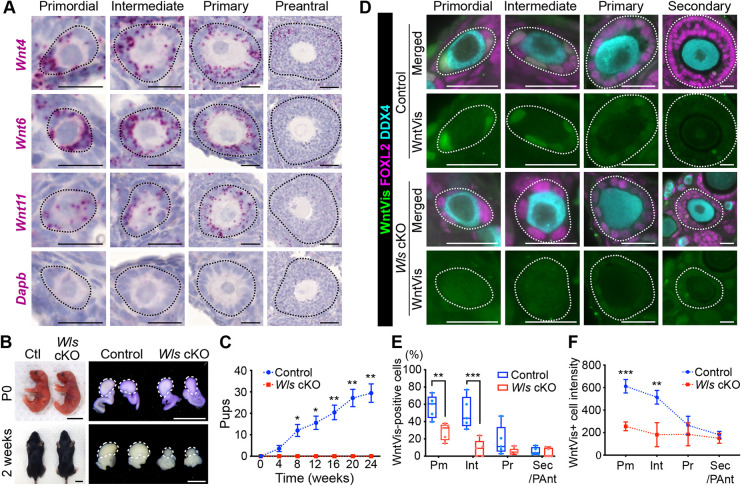
**Canonical WNT signaling in primordial follicles.** (A) *In situ* hybridization analysis of *Wnt4*, *Wnt6*, *Wnt11* and *Dapb* (negative control) mRNAs (red) in ovaries of 3-week-old wild-type (WT) mice. Follicles were classified as primordial, intermediate, primary or preantral (outlined by dotted lines). (B) Gross morphology of the body and ovaries (dotted lines) of *Wls* cKO and littermate control (Ctl) mice at P0 and 2 weeks of age. (C) Cumulative number of pups born to control or *Wls* cKO female mice housed with WT males for 24 weeks beginning at 8 weeks of age. Data are mean±s.e.m. (*n*=7 females of each genotype). *P<0.05, **P<0.01 (two-way ANOVA with Sidak's test for multiple comparisons). (D) Immunofluorescence staining of WntVis (green), FOXL2 (magenta) and DDX4 (cyan) in the ovaries of 3-week-old *Wls* cKO and littermate control mice harboring the *R26-WntVis* allele. White dotted lines outline follicles. (E) Percentage of WntVis-positive cells among pre-GCs/GCs for each follicle type as determined from images in D. Boxes indicate the median and 25th and 75th percentiles, the whiskers indicate minimum and maximum values (*n*=5 mice of each genotype). **P<0.01, ***P<0.001 (unpaired multiple *t*-tests with the Holm-Sidak correction). (F) Fluorescence intensity of WntVis-positive cells for each follicle type. Data are mean±95% confidence interval (*n*=725 cells from five control mice; *n*=256 cells from five *Wls* cKO mice). **P<0.01, ***P<0.001 (unpaired multiple *t*-tests with the Holm-Sidak correction). Pm, primordial; Int, intermediate; Pr, primary; Sec/PAnt, secondary/preantral. Scale bars: 50 μm (A, preantral); 20 μm (A, primordial, intermediate, primary; D); 1 cm (B, left); 100 μm (B, right).

Although WNT signaling has been implicated in adult folliculogenesis, the spatiotemporal patterns of WNT ligand expression in the mouse ovary have been insufficiently characterized (Harwood et al., 2008). Both the complexity and specificity of WNT signaling in mice are due in part to the expression of 19 WNT ligands. To identify the specific WNT ligands that are expressed during folliculogenesis, we performed *in situ* hybridization analysis with ovaries from 3-week-old wild-type (WT) mice for all 19 Wnt mRNAs (Fig. S1). Among the 19 WNT ligands, the mRNAs for *Wnt4*, *Wnt6* and *Wnt11* were detected in the GC lineage from the primordial follicle to primary follicle stages (Fig. 1A). The abundance of these WNT ligand mRNAs gradually declined in association with the transition to preantral follicles. Minimal expression of *Wnt2*, *Wnt2b*, *Wnt9a*, *Wnt5b*, *Wnt11* and *Wnt16* was observed in the oocytes of primordial follicles (Fig. 1A; Fig. S1). These findings suggest that pre-GC/GC-derived WNT signals might contribute to the early stages of folliculogenesis.

To examine the effects of attenuated WNT signaling, we generated ovarian somatic cell-specific *Wntless* conditional knockout (*Wls* cKO) mice by crossing *Sf1-Cre* mice [which express Cre recombinase under the control of the steroidogenic factor 1 gene (*Sf1*; *Nr5a1*) promoter] to mice harboring a ‘floxed’ (*Wls*^flox^) and a ubiquitous deletion (*Wls*^del^) allele of *Wls* (Carpenter et al., 2010; Dhillon et al., 2006). Given that WLS is required for secretion of all WNT ligands, the resulting *Sf1-Cre;Wls*^flox/del^ (*Wls* cKO) mice allow us to examine the effects of inhibiting WNT ligand secretion specifically from ovarian somatic cells, including GC and theca-lineage cells, from embryonic day (E) 11.5 (Dhillon et al., 2006; Piprek et al., 2019). *Sf1-Cre;Ai9* reporter mouse ovaries were used to compute Cre recombination efficiency in FOXL2-positive GC lineage cells, which was 98.30±0.72% at P0 and 97.37±1.08% at 2 weeks of age (mean±s.e.m.). We found no Cre activity in oocytes. In *Wls* cKO mice, no obvious morphological abnormalities were apparent during development through adulthood (Fig. 1B). To obtain *Wls* cKO mice, female *Wls*^flox/flox^ and male *Sf1-Cre;Wls*^del/+^ mice were mated. The male:female ratio of the resulting litters was 1.10 (*n*=500) overall and 1.14 (*n*=137) for the *Wls* cKO mice, with no significant difference using the chi-squared test. The birth rate of all *Wls* cKO mice was 27.4%, which is close to the theoretical rate of 25.0%. The ovaries of *Wls* cKO mice were similar to those of littermate control mice at postnatal day (P) 0, whereas they manifested atrophy at 2 weeks of age (Fig. 1B). To evaluate reproductive performance, we housed 8-week-old control or *Wls* cKO female mice (*n*=7 per genotype) with WT males for 24 weeks. *Wls* cKO females were completely infertile (Fig. 1C) even though they engaged in spontaneous mating behavior.

To identify WNT-responding cells, we evaluated the ovaries of a WNT signal reporter mouse line, *R26-WntVis*. The green fluorescent protein (GFP) reporter activity of these mice reflects the activity of the canonical WNT signaling pathway (Takemoto et al., 2016). GFP was specifically expressed in the GC lineage from the primordial to primary follicle stages (Fig. 1D), consistent with the expression pattern of Wnt mRNAs (Fig. 1A). FOXL2 was examined as a marker for pre-GCs/GCs and DDX4 as a marker for oocytes in this analysis. The WntVis signal was also sparsely detected in the interstitial cells, theca cells and ovarian epithelium, but not in blood vessels (Fig. S2A-E). It was undetectable in oocytes (Fig. 1D). The WntVis signals were most abundant and intense in pre-GCs of primordial follicles, and they became less abundant and less intense with follicle growth (Fig. 1D-F). In the control group, the median was 60.5% for WntVis-positive cells in the pre-GC population that were composed of primordial follicles (Fig. 1E). As primordial follicles contain several pre-GCs, it is expected that most primordial follicles are receiving Wnt signaling to some extent. Both the number of WntVis-positive pre-GCs/GCs and WntVis fluorescence intensity were significantly reduced in *Wls* cKO mice harboring the *R26-WntVis* allele compared with control mice (Fig. 1D-F). Together, these results thus suggested that autocrine WNT signaling activity in pre-GCs is required for female fertility.

### WNT signaling is required for the pre-GC to GC transition and subsequent development

**Fig. 2.**
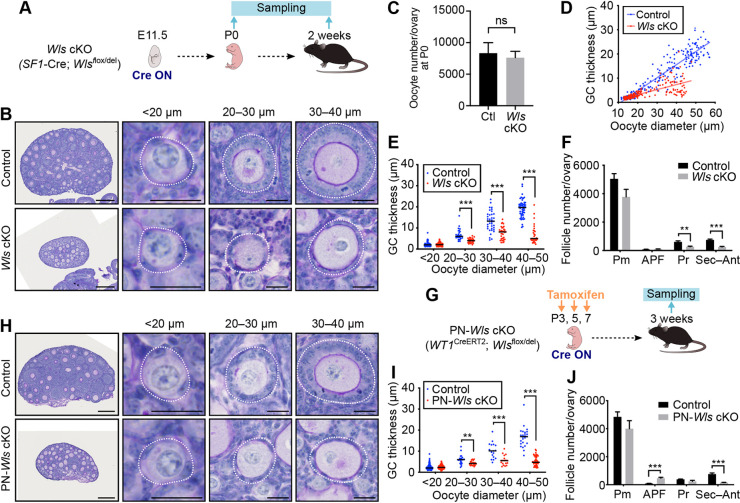
***Wls*** **cKO mice show an impaired transition of pre-GCs to GCs.** (A) Experimental scheme for examination of *Wls* cKO mice to determine the effects of embryonic deletion of *Wls* in ovarian somatic cells on folliculogenesis. (B) PAS-H staining of ovarian sections from 2-week-old *Wls* cKO mice. Follicles were classified by oocyte size (white dotted lines). (C) The number of DDX4-positive oocytes per ovary of *Wls* cKO or littermate control (Ctl) mice, as determined by immunohistochemical staining at P0. Data are mean+s.e.m. (*n*=7 mice of each genotype). ns, not significant (nonparametric Mann–Whitney matched-pairs test). (D) Scatter plot for the distribution of oocyte diameter and GC layer thickness for follicles of 2-week-old *Wls* cKO (*n*=200 follicles) and control (*n*=236 follicles) mice as determined from images similar to those in B. Regression lines are included. (E) GC layer thickness categorized by oocyte diameter for follicles of 2-week-old *Wls* cKO mice (*n*=208 follicles from four control mice; *n*=200 follicles from four *Wls* cKO mice). Horizontal lines represent the median. ***P<0.001 (unpaired multiple *t*-tests with the Holm-Sidak correction). (F) Quantification of follicle number per ovary for 2-week-old *Wls* cKO mice as determined by immunohistochemical staining for DDX4. Follicles were classified as primordial (Pm), activated primordial (APF: oocyte diameter of >20 μm without cuboidal GCs), primary (Pr) or secondary-antral (Sec-Ant). Data are mean+s.e.m. (*n*=7 mice per genotype). **P<0.01, ***P<0.001 (unpaired multiple *t*-tests with the Holm-Sidak correction). (G) Experimental scheme for examination of PN-*Wls* cKO mice to determine the effects of postnatal deletion of *Wls* on folliculogenesis in ovarian somatic cells. (H) PAS-H staining of ovarian sections from tamoxifen-treated 3-week-old PN-*Wls* cKO mice. (I) GC layer thickness categorized by oocyte diameter for follicles of 3-week-old PN-*Wls* cKO mice (*n*=239 follicles from six control mice; *n*=315 follicles from six PN-*Wls* cKO mice). Horizontal lines represent the median. **P<0.01, ***P<0.001 (unpaired multiple *t*-tests with the Holm-Sidak correction). (J) Quantification of follicle number per ovary for 3-week-old PN-*Wls* cKO mice as determined by immunohistochemical staining for DDX4. Data are mean+s.e.m. (*n*=7 mice per genotype). ***P<0.001 (unpaired multiple *t*-tests with the Holm-Sidak correction). Scale bars: 200 μm (B,H; leftmost panels); 20 μm (B,H; other panels).

**Fig. 3.**
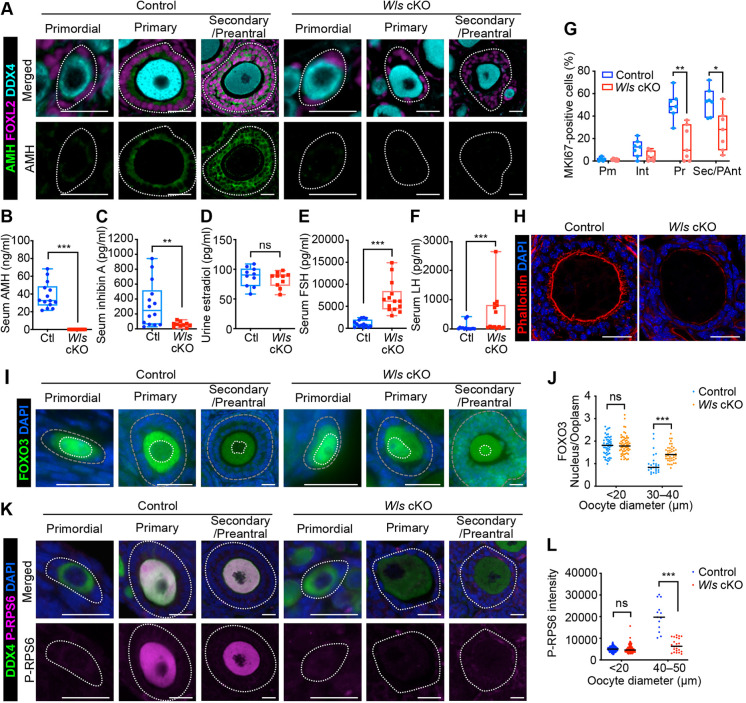
**Pre-GC to GC transition and oocyte activation are suppressed in *Wls* cKO mice.** (A) Immunostaining of AMH (green), DDX4 (cyan) and FOXL2 (magenta) in the ovaries of 3-week-old *Wl*s cKO or littermate control mice. Follicles are demarcated with white dotted lines. (B-F) Levels of AMH (B) and inhibin A (C) in serum, of estradiol in urine (D) and of FSH (E) and LH (F) in serum of 8-week-old *Wls* cKO and control (Ctl) mice [*n*=14 for control and *n*=10 for *Wls* cKO (B,C), *n*=10 (D), *n*=14 (E,F)]. The boxes indicate the median and 25th and 75th percentiles, and the whiskers represent minimum and maximum values. **P<0.01, ***P<0.001 (nonparametric Mann–Whitney matched-pairs test). ns, not significant. (G) Percentage of MKI67-positive pre-GCs/GCs for each follicle type (Pm, primordial; Int, intermediate; Pr, primary; Sec/PAnt, secondary/preantral) in 3-week-old *Wls* cKO and control mice (*n*=7) as determined by immunofluorescence staining. *P<0.05, **P<0.01 (unpaired multiple *t*-tests with the Holm-Sidak correction). (H) Staining of actin fibers with Phalloidin (red) and of nuclei with DAPI (blue) for growing follicles from 3-week-old *Wls* cKO or control mice. (I) Immunofluorescence staining of FOXO3 (green) for oocytes of 4-week-old *Wls* cKO or control mice. Nuclei were counterstained with DAPI (blue). White and gray dotted lines mark the boundaries of each oocyte nucleus and follicles, respectively. (J) The nucleus/cytoplasm ratio of FOXO3 fluorescence intensity in oocytes determined from images similar to those in I. Horizontal lines represent the median (*n*=80 oocytes from four control mice; *n*=102 oocytes from four *Wls* cKO mice). ***P<0.001 (unpaired multiple *t*-tests with the Holm-Sidak correction). ns, not significant. (K) Immunostaining of phospo-RPS6 (P-RPS6, magenta) and DDX4 (green) in the ovaries of 3-week-old *Wl*s cKO or littermate control mice. Nuclei were counterstained with DAPI (blue). Follicles are demarcated with white dotted lines. (L) Fluorescence intensity of P-RPS6 in oocytes determined from images similar to those in K. Horizontal lines represent the median (*n*=136 oocytes from four control mice; *n*=220 oocytes from five *Wls* cKO mice). ***P<0.001 (unpaired multiple *t*-tests with the Holm-Sidak correction). ns, not significant. Scale bars: 20 μm (A,I,K); 25 μm (H).

To investigate the cause of the ovarian defects of *Wls* cKO mice, we performed a more detailed morphological analysis (Fig. 2A). Immunostaining of DDX4 revealed that the number of oocytes per ovary did not differ significantly between *Wls* cKO and control mice at P0 (Fig. 2C). Periodic acid-Schiff staining with hematoxylin (PAS-H) revealed few atypical follicles, such as those containing multiple oocytes, in the ovaries of the mutant females at 2 weeks of age (Fig. 2B). Abnormal sexual differentiation was not apparent, as confirmed by sex genotyping (Fig. S3). These data suggested that germ cell survival during embryonic development and sex determination were not affected in *Wls* cKO mice. Whereas cuboidal GCs were apparent in growing follicles containing oocytes with a diameter of 20-40 μm in control mice, flattened and morphologically abnormal GCs were detected in *Wls* cKO mice (Fig. 2B). In contrast, no morphological abnormalities were detected in primordial follicles with an oocyte size of <20 μm (Fig. 2B). Quantitative analysis revealed that the GC layer was significantly thinner in growing follicles of *Wls* cKO mice, whereas it was similar in primordial follicles of both genotypes (Fig. 2D,E). GCs in *Wls* cKO mice were less likely to become multilayered, and even when they did form multiple layers, the layers were uneven (Figs 2B and 3A). To describe the morphology of GCs further, we categorized growing follicles by the appearance of GCs as squamous, cuboidal and columnar (Table S1). Within the growing follicles, primary follicles with squamous GCs were 26.2% in *Wls* cKO mice, notably higher than the 6.6% observed in controls. Secondary/preantral follicles with columnar GCs with distinct cell polarity were not observed in *Wls* cKO mice, but 48.4% were found in controls. Secondary/preantral follicles in *Wls* cKO mice mainly consisted of cuboidal type cells (27.9%). Overall, these results indicated that the PFA-associated transition of pre-GCs to GCs is suppressed in the absence of WNT signaling, yet GCs of some follicles can proceed to the cuboidal stage.

To assess whether WNT signaling might trigger PFA, we quantified the number of follicles per ovary and categorized them by follicle type as primordial (Pm), activated primordial (APF: oocyte diameter of >20 μm without cuboidal GCs), primary (Pr), or secondary-antral (Sec-Ant) at 2 weeks of age. The number of primordial follicles in *Wls* cKO mice was similar to that in control mice (Fig. 2F), suggesting that WNT ligands are not a triggering stimulus but rather a permissive signal for PFA; otherwise, the accumulation of primordial follicles in the mutant ovaries would have been expected. The observation that oocytes larger than 20 μm were present in the ovaries of *Wls* cKO mice (Fig. 2D,E) also suggested that these cells are capable of initiating a growth response to PFA. However, in contrast to control ovaries, the ovaries of 2-week-old *Wls* cKO mice lacked oocytes with a diameter of 45-60 μm. Oocytes with a diameter of >45 μm constituted 26.8±4.2% of all oocytes in control females but only 0.5±0.5% of those in *Wls* cKO females (*P*=0.0286, nonparametric Mann–Whitney matched-pairs test) (Fig. 2D). The retardation of oocyte growth in *Wls* cKO mice therefore appeared to occur between PFA and full maturity. The number of developing follicles was significantly lower in *Wls* cKO mice (Fig. 2F), with insufficient GC maturation likely giving rise to follicular atresia.

WNT signaling plays an important role in female sex determination during embryogenesis (Parma et al., 2006; Vainio et al., 1999). We therefore next examined the effects of postnatal deletion of *Wls* with the use of the *Wt1*^CreERT2^ knock-in allele (Zhou et al., 2008). Control and *Wt1*^CreERT2^;*Wls*^flox/del^ (PN-*Wls* cKO) mice were injected with tamoxifen at P3, P5 and P7 to induce *Wls*^flox^ deletion and were studied at 3 weeks of age (Fig. 2G). *Wt1*^CreERT2^;*Ai9* mice showed 99.5±0.09% (mean±s.e.m.) efficiency of the Cre recombination within FOXL2-positive cells at 3 weeks of age after tamoxifen administration. The phenotype of PN-*Wls* cKO female mice appeared to be essentially identical to that of *Wls* cKO females. The PN-*Wls* cKO mice thus showed morphologically normal primordial follicles and attenuated transition of pre-GCs to GCs in growing follicles (Fig. 2H,I). Primary follicles with squamous GCs made up 35.6% of the follicles counted in PN-Wls cKO mice whereas only 2.4% were found in controls at 3 weeks of age (Table S2). Hence, the defect found in the pre-GC to GC transition of *Wls* cKO and PN-*Wls* cKO mice is not the result of disrupted cell fate determination during embryogenesis, but rather a result from the lack of WNT signaling during folliculogenesis. The number of primordial follicles in PN-*Wls* cKO mice was also similar to that of control mice (Fig. 2J), providing further evidence that initiation of PFA can take place without WNT signaling. The ovaries of PN-*Wls* cKO mice also showed reduced numbers of growing follicles (Fig. 2J), reflecting suppression of folliculogenesis.

### Functional impairment of the transition of pre-GCs to GCs gives rise to insufficient oocyte activation

To evaluate whether GCs in *Wls* cKO mice are functionally mature, we assessed the expression of anti-Müllerian hormone (AMH), a marker for GCs. Pre-GCs of primordial follicles initially do not express AMH. AMH becomes expressed once GCs grow and transition to a cuboidal/columnar morphology and then is released into the circulation (Visser et al., 2006). In *Wls* cKO mice, however, immunofluorescence staining revealed only a low level of AMH expression in GCs (Fig. 3A). PFA is hypothesized to be a locally regulated process, whereas the later stages of folliculogenesis are influenced markedly by GC-derived paracrine factors and gonadotropins (Sánchez and Smitz, 2012). We therefore next analyzed major GC-derived hormones (AMH, inhibin A and estradiol) and gonadotropins [follicle-stimulating hormone (FSH) and luteinizing hormone (LH)] in order to shed light on GC function and the endocrine system in *Wls* cKO mice. The concentrations of AMH and inhibin A in serum were significantly lower in *Wls* cKO female mice than in controls at 8 weeks of age (Fig. 3B,C). These data indicated that GC function is markedly suppressed in *Wls* cKO mice. Estrogens are primarily produced by developing follicles to coordinate systemic reproductive functions (Hillier et al., 1994; Miller and Auchus, 2011), but in this study their urinary concentration did not differ between the two genotypes (Fig. 3D). Given that estrogen production to some extent has been reported in mutant mice lacking sexual maturation, ovariectomized mice and human patients whose ovarian steroidogenesis is inhibited (De Tassigny et al., 2007; Miller and Auchus, 2011; Saito et al., 2009), *Wls* cKO mice may also possess a compensatory mechanism that allows for estrogen production. By contrast, the serum levels of FSH and LH were significantly higher in *Wls* cKO mice than in control mice (Fig. 3E,F), possibly reflecting a positive feedback response to the suppressed follicle development and lack of ovulation in the mutant females. Pituitary gland function may thus be normal in *Wls* cKO females, even though *Sf1-Cre* is expressed in endocrine glands (Dhillon et al., 2006). Importantly, low Amh and high FSH levels in serum are considered diagnostic criteria for human POI (Jankowska, 2017; Méduri et al., 2007).

GC proliferation is a key contributor to follicle growth, and the mTOR signaling pathway, which is implicated in PFA, appears to regulate GC proliferation (Yu et al., 2011). To assess the role of other signaling pathways such as WNT signaling in GC proliferation, we examined *Wls* cKO mice by performing immunostaining for MKI67 (Ki67) and measuring the signal intensity for all FOXL2-positive pre-GCs/GCs within follicles. Although the percentage of MKI67-positive GCs increased with follicle growth in both control and *Wls* cKO mice, the increase was less pronounced in the mutant animals (Fig. 3G). Most pre-GCs of primordial follicles were negative for MKI67 in both control and *Wls* cKO mice (Fig. 3G). Transzonal projections (TZPs) are membranous extensions from GCs that pass through the zona pellucida to the oocyte cell membrane and are important for normal oocyte development (Albertini et al., 2001; Carabatsos et al., 1998). Staining of filamentous actin with Phalloidin revealed the absence of obvious TZP structures in *Wls* cKO ovaries (Fig. 3H). These results thus indicated that the abrogated folliculogenesis of *Wls* cKO mice is attributable to impaired GC proliferation and the inability of GCs to support oocyte growth.

Although we found that oocyte growth is initiated in *Wls* cKO mice, it was unclear whether the oocytes undergo normal activation. To evaluate oocyte status, we analyzed the expression of FOXO3, a transcription factor that contributes to maintenance of oocyte dormancy, by quantifying the nuclear to cytoplasmic ratio of its immunofluorescence intensity (Castrillon et al., 2003). In control mice, whereas primordial follicles manifested a nuclear FOXO3 localization, FOXO3 was exported from the nucleus during PFA (Fig. 3I,J). However, in *Wls* cKO mice, both oocytes with a diameter of <20 µm and those with a diameter of 30-40 μm showed a higher FOXO3 intensity in the nucleus than in the cytoplasm (Fig. 3J). In both primary and secondary follicles, FOXO3 was localized in the nuclei of growing oocytes of *Wls* cKO mice (Fig. 3I). FOXO3 is known to be phosphorylated by the PI3K-AKT pathway (John et al., 2008), whereas phosphorylation of the ribosomal protein S6 (RPS6) is a key downstream event of the PI3K-AKT-mTOR pathway in PFA (Adhikari et al., 2009; Reddy et al., 2008). RPS6 contributes to oocyte growth by promoting protein translation and ribosomal biogenesis. Phosphorylated-RPS6 is not obvious in the oocytes of primordial follicles, but becomes evident once they are activated. In *Wls* cKO mice, phosphorylated-RPS6 in activated oocytes is markedly suppressed (Fig. 3K,L). This result suggested that the delay in oocyte growth in *Wls* cKO mice (Fig. 2D) is likely due to insufficient function of RPS6. Collectively, these data indicated that, even if oocytes increase in size, they do not undergo the normal activation process in *Wls* cKO mice. These results further suggested that activated GCs are necessary for proper oocyte growth; the functions of FOXO3 and RPS6 via the overlapping PI3K-AKT pathway are under the influence of GCs. We then examined the expression levels of GC-derived KITL and its receptor KIT, which are thought to cause FOXO3 re-localization to the cytoplasm and contribute to oocyte activation (Liu et al., 2014; Saatcioglu et al., 2016). Contrary to our expectations, the expression of KITL and KIT was slightly increased in *Wls* cKO mice (Fig. S4A,B). How loss of WNT signaling might increase KIT and KITL is still a matter that needs further clarification. Nevertheless, the data suggest that, in addition to KIT signaling, unknown factors may act downstream of WNT signaling to impact FOXO3 activity.

### The pre-GC layer is expanded by a dominant stable form of CTNNB1

**Fig. 4.**
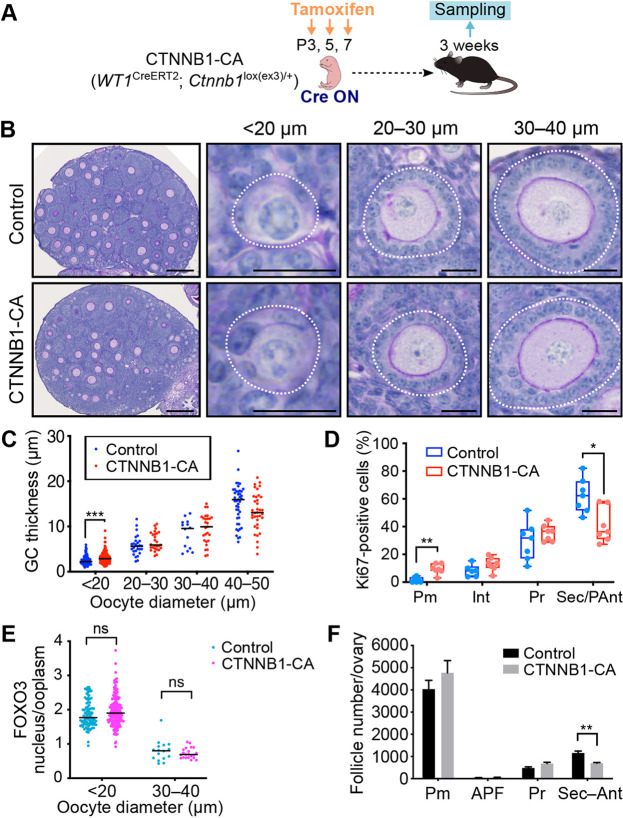
**Constitutively active CTNNB1 promotes the pre-GC to GC transition.** (A) Experimental scheme showing induction of a constitutively active form of CTNNB1 in ovarian somatic cells of CTNNB1-CA mice. (B) PAS-H staining of ovarian sections from 3-week-old (tamoxifen-treated) CTNNB1-CA and littermate control mice. Follicles (white dotted lines) were classified by oocyte size. (C) GC layer thickness categorized by oocyte diameter for 3-week-old CTNNB1-CA and control mice. Horizontal lines represent the median (*n*=226 follicles from five control mice; *n*=224 follicles from five CTNNB1-CA mice). ***P<0.001 (unpaired multiple *t*-tests with the Holm-Sidak correction). (D) Percentage of MKI67-positive pre-GCs/GCs for each follicle type (Pm, primordial; Int, intermediate; Pr, primary; Sec/PAnt, secondary/preantral) of 3-week-old CTNNB1-CA and control mice as determined by immunofluorescence staining. Boxes indicate the median and 25th and 75th percentiles; whiskers represent minimum and maximum values (*n*=7 mice per genotype). *P<0.05, **P<0.01 (unpaired multiple *t*-tests with the Holm-Sidak correction). (E) The nucleus/cytoplasm ratio of FOXO3 immunofluorescence intensity for oocytes of 3-week-old CTNNB1-CA and control mice. Horizontal lines represent the median (*n*=110 oocytes from four control mice; *n*=216 oocytes from five CTNNB1-CA mice). ns, not significant (unpaired multiple *t*-tests with the Holm-Sidak correction). (F) Quantification of follicle number per ovary for 3-week-old CTNNB1-CA and control mice. Follicles were classified as primordial (Pm), activated primordial (APF), primary (Pr) or secondary–antral (Sec–Ant). Data are mean+s.e.m. (*n*=7 mice per genotype). **P<0.01 (unpaired multiple *t*-tests with the Holm-Sidak correction). Scale bars: 200 μm (A, leftmost panels); 20 μm (A, other panels).

To investigate whether WNT signaling is sufficient for the pre-GC transition to GCs, we generated *Wt1*^CreERT2^;*Ctnnb1*^lox(ex3)/+^ (CTNNB1-CA) mice, which express a stable form of CTNNB1 in the somatic lineage of ovaries (Fig. 4A). The stabilized CTNNB1 binds to T cell factor/lymphoid enhancer factor (TCF/LEF) transcription factors, which activate the expression of target genes for canonical WNT signaling (Harada et al., 1999). Ovaries of (tamoxifen-treated) CTNNB1-CA mice were similar in size to those of control mice at 3 weeks of age, but were more spherical in appearance and had a smoother surface compared with control ovaries (Fig. 4B). The observation that somatic cells were densely packed in the ovarian interstitium of CTNNB1-CA mice suggests that hyperproliferation of interstitial cells was responsible for these differences. Morphological abnormalities were not apparent in GCs of growing follicles in the mutant mice, whereas pre-GCs of primordial follicles were not squamous but cuboidal (Fig. 4B). An increase in pre-GC layer thickness was also detected in primordial follicles containing oocytes with a diameter of <20 μm in CTNNB1-CA mice (Fig. 4C), and MKI67 immunostaining revealed that the proliferation of pre-GCs in primordial follicles was enhanced (Fig. 4D). Follicles with oocytes of <20 μm and with four or fewer pre-GCs/GCs that showed no obvious columnar shape were classified as primordial follicles in the mutant ovaries. These results thus revealed that Wnt signaling promotes the transition of pre-GCs to GCs. Of note, CTNNB1-CA mice showed a normal subcellular localization pattern for FOXO3 in their oocytes (Fig. 4E). Given that we believe that WNT plays only a permissive role in PFA, it was not surprising that localization of FOXO3 in oocytes was not affected by hyperactivation of canonical WNT signaling in GCs. However, the expression levels of KITL and KIT were slightly reduced in CTNNB1-CA (Fig. S4C), in contrast to the *Wls* cKO phenotypes. As WNT and KIT signaling are fundamental pathways to regulate the pre-GC transition to GC and oocyte growth, respectively, it is possible that these two pathways can mutually adjust their activity in order to balance the PFA outcome, an idea that can be tested in future studies.

Quantification of follicle number revealed no depletion of primordial follicles or increase in the number of developing follicles in CTNNB1-CA mice (Fig. 4F), suggesting that CTNNB1 stabilization (activation of canonical WNT signaling) is insufficient for induction of PFA. Inhibition of follicle growth was apparent in the mutant mice, however, with the number of secondary/preantral follicles being significantly reduced (Fig. 4F), and GCs secondary/preantral follicles were less proliferative (Fig. 4D). Constitutive activation of WNT signaling likely influences GCs, interstitial cells and theca cells in such a manner that the survival and growth of growing follicles are impaired. These characteristics are consistent with the reduced proliferative capacity and cancerous changes of GCs previously observed for mice in which CTNNB1 or the WNT agonist RSPO1 was forcibly expressed (Boerboom et al., 2005, 2006; De Cian et al., 2017).

### A WNT activator rescues the phenotype of *Wls* cKO mouse ovaries *in vitro*

**Fig. 5.**
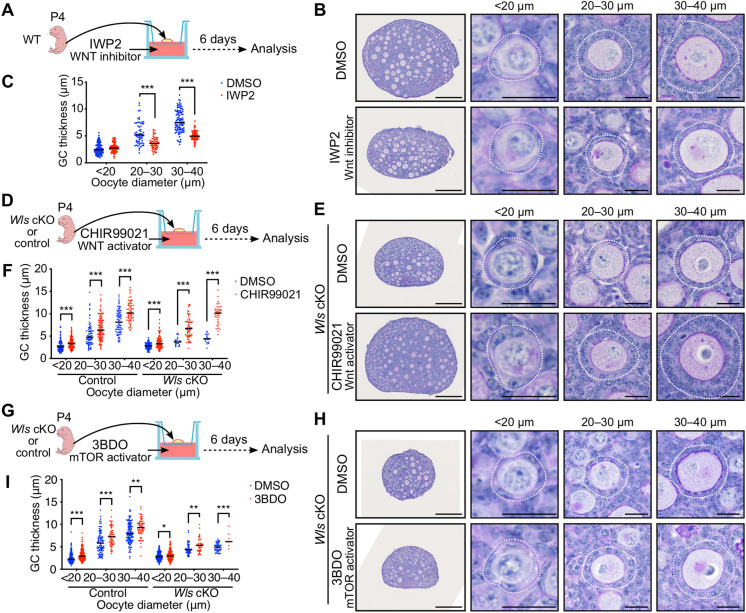
**Rescue of the *Wls* cKO ovarian phenotype by a WNT activator *in vitro*.** (A,D,G) Experimental design for culture of ovaries from the indicated mice with the WNT inhibitor IWP2 at 2 μM (A), the WNT activator CHIR99021 at 5 μM (D) or the mTOR signaling activator 3BDO at 100 μM (G) or with the corresponding concentration of DMSO as a vehicle control. (B,E,H) PAS-H staining of sections of ovaries cultured for 6 days with IWP2 (B), CHIR99021 (E) or 3BDO (H). Follicles were classified by oocyte diameter and are demarcated by the white dotted lines. (C,F,I) GC layer thickness categorized by oocyte diameter for ovaries cultured with IWP2 (C), CHIR99021 (F) or 3BDO (I). Horizontal lines represent the median [DMSO, *n*=280; IWP2, *n*=220 (C). Control+DMSO, *n*=245; control+CHIR99021, *n*=348; *Wl*s cKO+DMSO, *n*=162; *Wl*s cKO+CHIR99021, *n*=223 (F). Control+DMSO, *n*=457; control+3BDO, *n*=330; *Wl*s cKO+DMSO, *n*=360; *Wl*s cKO+3BDO, *n*=274 (I)]. *P<0.05, **P<0.01, ***P<0.001 (unpaired multiple *t*-tests with the Holm-Sidak correction). Scale bars: 200 μm (leftmost panels); 20 μm (other panels).

To verify the phenotype of *Wls* cKO mice, we determined the effects of a WNT inhibitor in ovarian culture. Ovaries isolated from WT mice at P4 were maintained on membrane cell culture inserts for 6 days by the gas-liquid interphase method, either in the presence of dimethyl sulfoxide (DMSO) as a vehicle control or the WNT inhibitor IWP2, which blocks porcupine O-acyltransferase (PORCN)-mediated palmitoylation and consequent secretion of WNT ligands (Chen et al., 2009) (Fig. 5A). The ovaries at the end of the culture period thus corresponded to ovaries at P10 *in vivo*. PAS-H staining revealed that IWP2 markedly suppressed GC layer development, whereas it had only a minimal effect on primordial follicles with an oocyte diameter of <20 μm (Fig. 5B,C). We then cultured ovaries from *Wls* cKO or control mice with the WNT activator CHIR99021 in an attempt to rescue the phenotype of the mutant ovaries (Fig. 5D). CHIR99021 activates the canonical WNT signaling pathway by inhibiting glycogen synthase kinase 3 (GSK3) and thereby stabilizing CTNNB1 (Bennett et al., 2002). CHIR99021 induced a significant thickening of the GC layer at all assessed follicular stages in both control and *Wls* cKO ovaries (Fig. 5E,F). Of note, the abnormal flattened morphology of GCs in *Wls* cKO ovaries was completely normalized by CHIR99021 treatment (Fig. 5E). These data indicated that the function of WNT signaling in folliculogenesis was evident *in vitro*, and that a WNT activator was able to promote follicle growth in a commonly adopted culture system.

The mTOR signaling pathway is implicated in PFA. Nutritional or other factors are thus thought to activate mTOR signaling in pre-GCs and thereby stimulate the production of KITL required for oocyte activation (Liu et al., 2014). Given that WNT signaling has been shown to activate mTOR complex 1 (mTORC1) as a result of inhibition of GSK3 (Inoki et al., 2006), we investigated the potential role of WNT signaling as an upstream regulator of mTOR signaling in GCs. The addition of an activator of mTOR signaling, 3BDO, to ovarian cultures induced a significant increase in GC layer thickness in follicles of *Wls* cKO and control mice (Fig. 5G-I). However, this rescue effect for *Wls* cKO ovaries was limited, even in growing follicles (Fig. 5H,I). These data suggested that WNT and mTOR signaling contribute to PFA in a coordinated manner, rather than through a simple hierarchical relationship.

## DISCUSSION

**Fig. 6.**
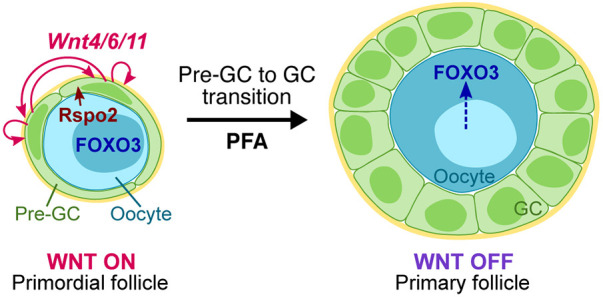
**Proposed role of WNT signaling in folliculogenesis.** WNT-responding pre-GCs produce *Wnt4*, *Wnt6* and *Wnt11* in primordial follicles and oocytes secrete the WNT agonist RSPO2. Activation of canonical WNT signaling in pre-GCs promotes their transition into GCs during primordial follicle activation (PFA). In primary follicles, GCs induce the withdrawal of oocytes from a dormant state, as reflected by the translocation of FOXO3 from the nucleus to the cytoplasm.

In this study, we propose a postnatal function of canonical WNT signaling to permit the transition of pre-GCs to GCs in an autocrine manner, which is required for facilitating oocyte growth (Fig. 6). Without WNT signaling, the majority of the GC population manifested characteristics similar to pre-GCs, including a squamous shape, hypoproliferative state, limited production of AMH and lack of TZP formation. WNT-mediated pre-GC to GC transition appears to couple PFA with the nuclear-cytoplasmic shuttling of FOXO3 and phosphorylation of RPS6 in oocytes to exit from dormancy. As neither attenuation nor enhancement of WNT signaling influences the number of remaining primordial follicles, we hypothesize that the initial trigger for PFA involves other stimuli, but that WNTs are crucial regulators of GC activation. Given that WntVis signals were undetectable in oocytes, our data do not support the possibility that oocytes directly respond to WNT ligands produced in GCs by activating the canonical WNT signaling pathway, although further studies will be required to fully exclude this possibility. Our findings thus highlight the importance of GC-oocyte communication for functional follicle growth and fertility, taking into consideration the fact that oocytes are able to complete maturation and attain their full size only with the support of GCs, the activation of which is dependent on WNT signaling.

Our results reveal that WNT signal activation occurs exclusively at the primordial follicle stage. WNT signaling in cuboidal/columnar GCs is likely detrimental to folliculogenesis, given that forced activation of canonical WNT signaling in pre-GCs/GCs reduced the number of developing follicles. Our data are consistent with the previous finding that activation of WNT signaling induced abnormal follicle growth with increased GC apoptosis in an *in vitro* culture of secondary follicles (Li et al., 2014). The activation of WNT signaling specifically at the primordial follicle stage is likely achieved as a result of the characteristic expression pattern of *Wnt4*/*6*/*11*, which is strongly expressed in the primordial to primary follicle stage, followed by a weaker expression in GCs of preantral follicles as they grow. Because WNT signaling activation occurs during a narrower time window than when the Wnt ligands are expressed, additional mechanisms may regulate the timing of WNT signaling activation. For example, production of functional RSPO2 by oocytes is important for the activation of canonical WNT signaling in GCs (De Cian et al., 2020). Given that *Rspo2* mRNA was found to be abundant in oocytes of growing follicles, a mechanism likely exists to inhibit WNT signaling after the primary follicle stage. BMP15 has been identified as an inhibitor of WNT signaling during early embryogenesis in *Xenopus* (Di Pasquale and Brivanlou, 2009), and activated mouse oocytes begin to secrete BMP15 at the primary follicle stage (Dube et al., 1998), which makes BMP15 a candidate mediator of WNT signaling in growing follicles. WNT signaling in pre-GCs/GCs may thus be precisely controlled at several levels, including the spatiotemporal specificity of Wnt ligand expression and the production of RSPO2 and BMP15 by oocytes.

It was reported that WNT4/RSPO1 initiates ovarian differentiation by activating the canonical WNT signaling pathway at the embryonic stage (Chassot et al., 2014). *Wnt4* is expressed in the gonads of both sexes until E11.5; it is then repressed in the male gonads and becomes ovary-specific from E12.5 onward. Although partial gonadal sex reversal has been reported in *Wnt4* and *Rspo1* null mice (Parma et al., 2006; Vainio et al., 1999), no such phenotype was observed in the *Wls* cKO mice used in this study. It is possible that the suppression of WNT secretion may have occurred later than gonadal sex determination, as there is likely a delay between the initial expression of Cre recombinase, the deletion of the target *Wls* gene and the suppression of WNT protein secretion. As SF1 expression in somatic cells of female gonads is known to be attenuated after E12.5 (Ikeda et al., 1994), *Wls* cKO under *Sf1-Cre* control may have progressed gradually until birth. It should be noted that the Cre expression starts from E11.5 in the *Sf1-Cre* line we used (Dhillon et al., 2006; Piprek et al., 2019), whereas another *Sf1-Cre*, which is often used in studies of sex differentiation, is reported to be expressed from E10.5 onward (Bingham et al., 2006). Recently Cheng et al. reported GC-specific conditional deletion of *Wls* by using *Amhr2-Cre* that initiates Cre expression from E13.5 (Cheng et al., 2020). They reported impaired luteinization of *Amhr2-Cre*; *Wls*^flox/flox^ mice, although no abnormalities in sex determination were noticed, which is consistent with the present study. It is possible that Cre-mediated recombination of the *Wls* gene may not be highly efficient, which might explain the absence of sex reversal and the phenotypic difference between *Amhr2-Cre*; *Wls*^flox/flox^ (homozygous for flox) and *Sf1-Cre;Wls*^flox/del^ (heterozygous for flox and null). We successfully showed the role of postnatal WNT signaling by generating PN-*Wls* cKO mice, but the fetal phenotype of *Wls* cKO ovaries needs to be scrutinized further. As *Sf1-Cre* is expressed in the theca cell lineage as well as pre-GC/GCs, the potential role of theca cell-derived WNTs on pre-GC development and PFA should be addressed in follow-up studies using theca cell-specific Cre mice.

Recent progress in the field of *in vitro* gametogenesis (IVG) has had a great impact on reproductive biology and medicine (Hikabe et al., 2016; Hamazaki et al., 2021). Fully developed oocytes can now be obtained from embryonic stem cells or induced pluripotent stem cells of mice by the application of IVG techniques. Although the protocol for stem cell-derived oocytes for fertility treatment in humans has not yet been fully established, vigorous research is underway (Yamashiro et al., 2018). Simultaneously, *in vitro* activation (IVA) has recently been described as an innovative method of fertility treatment for women with POI (Kawamura et al., 2013; Suzuki et al., 2015; Zhai et al., 2016). Wnt-related genes have not been identified as genes responsible for POI, but we have now shown that the hormonal environment of *Wls* cKO mice is similar to that of women with POI (De Vos et al., 2010; Jankowska, 2017). This finding suggests that some cases of POI diagnosed as idiopathic may include those attributable to insufficient transition of pre-GCs to GCs. In the IVA method, the oocyte-awakening process (PTEN-PI3K-AKT-FOXO3 signaling) is targeted in order to activate the few remaining primordial follicles. This method is applicable not only to POI patients but also to cancer patients whose only option for having children with their own oocytes is to cryopreserve their ovaries. However, it is hoped that this method will be developed further, because of the 7.8% chance of pregnancy yielded by IVA treatment (Kawamura et al., 2013; Suzuki et al., 2015; Zhai et al., 2016), which is comparable to the estimated 5-10% chance of pregnancy in POI patients (Van Kasteren and Schoemaker, 1999). In this study, treatment of ovarian cultures with the WNT activator CHIR99021 increased the thickness of the GC layer in early developing follicles (oocyte size of 20-40 µm). It is important to note that such a scenario was not observed in response to activation of WNT signaling in CTNNB1-CA mice; however, this difference may be due to a difference in the extent of WNT signaling activation, or to an effect of CHIR99021 on cell survival (Wang et al., 2015). Given that our study demonstrates that a WNT activator induced GC layer thickening and enhanced follicle growth *in vitro*, transient administration of a WNT activator such as CHIR99021 or WNT proteins on IVA may prove to be clinically beneficial for enhancing the pre-GC transition to GC and thereby to lead efficient PFA and successful pregnancy.

## MATERIALS AND METHODS

### Animals

*Sf1-Cre* mice (stock no. 012462), *Wls*^flox^ mice (stock no. 012888), *Wt1*^CreERT2^ mice (stock no. 010912), *Ddx4-Cre* mice (stock no. 006954) and *Ai9* mice (stock no. 007909) were obtained from The Jackson Laboratory (Carpenter et al., 2010; Dhillon et al., 2006; Gallardo et al., 2007; Madisen et al., 2010; Zhou et al., 2008). *Wls*^del^ mice, in which the *Wls*^flox^ allele is deleted ubiquitously, were generated by crossing *Wls*^flox^ mice with *Ddx4-Cre* mice. *R26-WntVis* mice (accession no. CDB0303 K) were obtained from the Laboratory for Animal Resources and Genetic Engineering at the RIKEN Center for Biosystems Dynamics Research, Kobe, Japan (http://www.clst.riken.jp/arg/reporter_mice.html) (Takemoto et al., 2016). *Ctnnb1*^lox(ex3)^ mice were kindly provided by M. M. Taketo (Kyoto University, Japan) (Harada et al., 1999). *Sf1-Cre;Wls*^flox/+^ and *Wls*^flox/del^ mice were used as littermate controls for *Sf1-Cre;Wls*^flox/del^ (*Wls* cKO) mice. Tamoxifen-injected *Wt1*^CreERT2^;*Wls*^flox/+^ and *Wls*^flox/del^ mice were used as littermate controls for *Wt1*^CreERT2^;*Wls*^flox/del^ (PN-*Wls* cKO) mice. Tamoxifen-injected *Wt1*^CreERT2^ and *Ctnnb1*^lox(ex3)/+^ mice were used for littermate control for *Wt1*^CreERT2^;*Ctnnb1*^lox(ex3)/+^ (CTNNB1-CA) mice. Tamoxifen (0.2 mg per 20 g of body weight) was injected intraperitoneally into mice at P3, P5 and P7. All animal experiments were approved by the Institutional Animal Care and Use Committee of RIKEN (approval number: A2017-13-5). All mouse lines studied were maintained on a mixed genetic background.

### Fertility test

Eight-week-old control or *Wls* cKO female mice (*n*=7 for each genotype) were housed continuously with WT (C57BL/6N) males for 24 weeks, and the numbers of pups produced were counted.

### *In situ* hybridization

*In situ* hybridization was performed with the use of the RNAscope system (Wang et al., 2012). Ovaries from 3-week-old WT mice were fixed in 10% neutral buffered formalin at room temperature for 24 h, and then dehydrated and embedded in paraffin. Tissue sections were processed for *in situ* detection of RNA with the RNAscope 2.5 High Definition (HD)-Red Assay (ACDBio). All probes were purchased from ACDBio: *Dapb* (310043, EF191515, target region 414-862), *Wnt4* (401101, NM_009523.2, target region 2147-3150), *Wnt6* (401111, NM_009526.3, target region 780-2026), *Wnt11* (405021, NM_009519.2, target region 818-1643), *Polr2a* (312471, NM_009089.2, target region 2802-3678), *Wnt1* (401091, NM_021279.4, target region 1204-2325), *Wnt2* (313601, NM_023653.5, target region 857-2086), *Wnt2b* (405031, accession no. NM_009520.3, target region 1307-2441), *Wnt3* (312241, NM_009521.2, target region 134-1577), *Wnt3a* (405041, NM_009522.2, target region 667-1634), *Wnt5a* (316791, NM_009524.3, target region 200-1431), *Wnt5b* (405051, NM_001271757.1, target region 319-1807), *Wnt7a* (401121, NM_ 009527.3, target region 1811-3013), *Wnt7b* (401131, NM_009528.3, target region 1597-2839), *Wnt8a* (405061, NM_009290.2, target region 180-1458), *Wnt8b* (405071, NM_011720.3, target region 2279-3217), *Wnt9a* (405081, NM_139298.2, target region 1546-2495), *Wnt9b* (405091, NM_011719.4, target region 706-1637), *Wnt10a* (401061, NM_009518.2, target region 479-1948), *Wnt10b* (401071, NM_011718.2, target region 989-2133), and *Wnt16* (401081, NM_053116.4, target region 453-1635).

### Immunostaining and histology

Immunofluorescence staining was performed with ovaries that had been fixed overnight at 4°C with 4% paraformaldehyde in phosphate-buffered saline (PBS). The fixed tissue was dehydrated, embedded in paraffin and then sectioned at a thickness of 5 μm. The sections were depleted of paraffin by immersion in xylene 3 times for 10 min and rehydrated through an alcohol series (twice in 100%, 95% and 85% ethanol for 1 min in each, 20 min in 70% ethanol, and then twice for 5 min in distilled water). For antigen retrieval, they were incubated either at 110°C for 15 min with citrate buffer (pH 6.0) or at 90°C for 20 min with HistoVT One (Nacalai Tesque). After washing with PBS containing 0.1% Tween 20 (PBST), the sections were incubated for 1 h at room temperature in a blocking buffer, stained overnight at 4°C with primary antibodies, and then exposed for 2 h at room temperature to a 1:500 dilution of secondary antibodies labeled with Alexa Fluor 488, 568, or 647 (A11057, A-21206, A21447, A21463 and A10042, Thermo Fisher Scientific; or 703-545-155, Jackson ImmunoResearch). The primary antibodies included chicken anti-GFP (1:500; GFP-1010, Aves Labs), rabbit anti-DDX4 (1:500; ab13840, Abcam), mouse anti-DDX4 (1:200; ab27591, Abcam), goat anti-FOXL2 (1:200; ab5096, Abcam), rabbit anti-AMH (1:100; GTX129593, GeneTex), rabbit anti-FOXO3 (1:500; 12829, Cell Signaling Technology), rat anti-MKI67 (1:400; 14-5698-82, eBioscience), rabbit anti-phospho-RPS6 (1:100, 4857, Cell Signaling Technology), goat anti-KIT (1:100, AF1356, R&D Systems) and rabbit anti-KITL (1:40, ab64677, Abcam). Antibodies were diluted in blocking buffer or Can Get Signal immunostain solutions (NKB-401, Toyobo). DNA was counterstained with 4′,6-diamidino-2-phenylindole (DAPI). Samples were mounted with Vectashield Vibrance Antifade Mounting Medium (H-1700, Vector Laboratories).

For immunofluorescence staining of GFP, CYP17A1 and PECAM1, ovaries were fixed overnight at 4°C with 4% paraformaldehyde in PBS. The fixed tissue was immersed in sucrose gradients (10%, 20% and 30%) in PBS sequentially at 4°C; then, tissues were embedded in Tissue-Tek OCT Compound (Sakura Finetek). Frozen samples were sectioned at 6 μm using CryoStar NX70 (Leica Microsystems). For antigen retrieval, cryosections were incubated at 70°C for 30 min with HistoVT One (Nacalai Tesque). After washing with PBST, the sections were incubated in blocking buffer for 1 h at room temperature, stained overnight at 4°C with primary antibodies, and then exposed for 2 h at room temperature to a 1:500 dilution of secondary antibodies labeled with Alexa Fluor 488, 568 or Dylight 650 (A10042 and SA5-10029, Thermo Fisher Scientific; or 703-545-155, Jackson ImmunoResearch). The following primary antibodies were used: chicken anti-GFP (1:500; GFP-1010, Aves Labs), rabbit anti-CYP17A1 (1:4000, 14447-1-AP, Proteintech) and rat anti-PECAM1 (1:100, sc-18916, Santa Cruz Biotechnology). These were diluted in Can Get Signal immunostain solutions (Toyobo). DNA was counterstained with DAPI. Samples were then mounted with Vectashield Vibrance Antifade Mounting Medium.

For Phalloidin staining, ovaries fixed with 4% paraformaldehyde in PBS were embedded in Tissue-Tek OCT Compound (Sakura Finetek) and cryosectioned at a thickness of 6 μm. The sections were washed with PBST, stained with Alexa Fluor 568-conjugated Phalloidin (1:100; A12380, Thermo Fisher Scientific) and DAPI, and then mounted with Vectashield Vibrance Antifade Mounting Medium.

For Cre recombination efficiency measurements, 6 μm ovarian cryosections of *Sf1-Cre;Ai9* or tamoxifen-injected *Wt1*^CreERT2^;*Ai9* mice were treated with goat anti-FOXL2 (1:100; ab5096, Abcam) and subsequently with donkey anti-goat IgG Alexa Fluor 647 (1:500; A21447, Thermo Fisher Scientific), and then mounted with Vectashield Vibrance Antifade Mounting Medium.

For PAS-H staining, ovaries were fixed in Bouin's solution, embedded in paraffin and sectioned at a thickness of 5 μm. The sections were depleted of paraffin by immersion in xylene three times for 10 min and rehydrated through an alcohol series (twice in 100%, 95% and 85% ethanol for 1 min in each, 20 min in 70% ethanol and twice for 5 min in distilled water). Next, the sections were treated with 0.5% periodic acid solution for 10 min, then stained with Schiff's reagent for 15 min. The staining reaction was stopped by treatment with a sulfurous acid solution three times for 2 min, followed by Hematoxylin counterstaining for 2 min. The sections were rinsed with tap water twice after each step for 5 min.

For quantitative analysis of follicle numbers, Bouin's fixed ovarian sections were immunostained for DDX4. Ovaries fixed with Bouin's solution were embedded in paraffin, serially sectioned at a thickness of 8 μm and then hydrated. After treatment with EDTA buffer (pH 8.0) at 110°C for 15 min, every fifth section was incubated consecutively with antibodies to DDX4 (1:500; ab13840, Abcam) and biotinylated secondary antibodies (1:500; BA-1000, Vector Laboratories). Immune complexes were detected using a Streptavidin Biotin Complex Peroxidase Kit (30462-30, Nacalai Tesque) and Peroxidase Stain DAB Kit (25985-50, Nacalai Tesque). Nuclei were counterstained with Hematoxylin.

### Image analysis

Immunostaining was examined using a BX53 upright microscope (Olympus) or a slide scanner (Axio Scan.Z1, Zeiss). Phalloidin staining was examined using a confocal laser scanning microscope (TCS SP8, Leica Microsystems). *In situ* hybridization and PAS-H staining were examined using a slide scanner (Axio Scan.Z1, Zeiss). All images taken with Axio Scan.Z1 utilized the tile scan and automated stitching functions.

For measurement of Cre recombination efficiency, ovarian sections of *Sf1-Cre;Ai9* or *Wt1*^CreERT2^;*Ai9* mice immunostained for FOXL2 were used. Littermate *Ai9* mice were used as negative controls. Images were acquired with a slide scanner (Axio Scan.Z1, Zeiss). The presence or absence of tdTomato fluorescence in FOXL2-positive cells was manually identified on randomly selected ovarian sections from three individuals in each group.

For measurement of WntVis or MKI67 signals in pre-GCs/GCs, ovarian sections were subjected to immunofluorescence staining for GFP or MKI67, respectively, as well as for the GC marker FOXL2 and the oocyte marker DDX4. With the use of ImageJ software (National Institutes of Health), areas positive for both FOXL2 and DAPI were determined as nuclear regions of GCs. The fluorescence intensity of GFP or MKI67 in each region was measured. The lower thresholds for GFP- or MKI67-positive cells were set at the value with 99% accuracy in the negative control samples. More than five ovaries for each genotype as well as more than one section per ovary were analyzed. Results were summarized according to follicle type: primordial, intermediate (containing an oocyte surrounded by a mixed single layer of pre-GCs and GCs), primary, and secondary/preantral. Only follicles with a visible nucleolus in the oocyte were analyzed.

For quantitative analysis of follicle numbers, Bouin's fixed ovarian sections with immunostaining for DDX4 were analyzed. Only follicles with a visible nucleolus in the oocyte were counted. The raw counts of follicle number were multiplied by five to account for the unanalyzed sections and to obtain the estimates of follicle number per ovary. The follicles were classified into primordial follicles (containing an oocyte with a diameter of <20 μm and surrounded by flat pre-GCs), activated primordial follicles (containing an oocyte with a diameter of >20 μm but not containing cuboidal GCs), primary follicles (containing an oocyte surrounded by a single layer of cuboidal GCs), secondary/preantral follicles (containing an oocyte surrounded by two or more layers of GCs), and antral follicles (containing an oocyte surrounded by multilayered GCs with antral cavity). Seven ovaries for each genotype were analyzed.

For quantification of the subcellular localization of FOXO3, images of immunostained ovarian sections for FOXO3 were analyzed using ImageJ. The fluorescence intensity of FOXO3 in cytoplasmic and nuclear (DAPI-positive) regions of oocytes was measured together with oocyte diameter. The nuclear to cytoplasmic ratio of FOXO3 intensity was then determined. More than five ovaries for each genotype, and more than one section per ovary, were analyzed.

For the quantification of phospo-RPS6, KIT or KITL intensities, images of ovarian sections immunostained for phospo-RPS6, KIT or KITL were analyzed using ImageJ in the oocyte or the GC area. The regions of GC and oocyte were determined manually using ImageJ. More than four ovaries for each genotype, and one section per ovary, were analyzed.

The thickness of the GC layer was determined as half the difference between the diameters of the follicle and the oocyte as measured in PAS-H-stained ovarian sections using ImageJ. More than four ovaries for each genotype or treatment, and more than one section per ovary, were analyzed.

For detailed morphometric analyses of growing follicles, primary and secondary follicles were categorized by the appearances of GCs on PAS-H stained Bouin's fixed sections as follows: squamous: more than 70% of GCs are squamous. The aspect ratio of GCs is approximately 2.0-4.0. Squamous/cuboidal: a mixture of squamous and cuboidal cells or GCs show the intermediate feature of squamous and cuboidal. Cuboidal: more than 70% of GCs are cuboidal. The cell aspect ratio is approximately 0.8 to 1.2. Cuboidal/columnar: a mixture of cuboidal and columnar cells or GCs show the intermediate feature of cuboidal and columnar cells. Columnar: nuclei are close to one side of the cytoplasm and show apparent cell polarity. The aspect ratio of GCs is approximately 0.6-0.8. Only follicles with a visible nucleolus in the oocyte were counted in randomly selected sections. More than four ovaries for each genotype, and one section per ovary, were analyzed.

### Sex genotyping

For genotyping, we used tail or toe (newborn pups only) snips. The genomic region of *Uba1* was amplified by PCR using Phire Hot Start II DNA Polymerase (Thermo Fisher Scientific). A primer set to detect *Uba1* (5′-TGGTCTGGACCCAAACGCTGTCCACA-3′ and 5′-GGCAGCAGCCATCACATAATCCAGATG-3′) was used to determine the chromosomal sexes, as described in a previous report (Chuma and Nakatsuji, 2001). The PCR products were separated by 3.0% agarose gel electrophoresis.

### Measurement of hormone levels

Mice at random stages of the estrous cycle were anesthetized using isoflurane before cardiac puncture for blood collection, and they were then euthanized by cervical dislocation. Collected blood was allowed to clot at room temperature for at least 30 min before centrifugation at 10,000 ***g*** for 5 min for serum extraction. For collection of urine, mice were manually restrained and allowed to urinate on disposable plastic trays. Serum and urine were stored at −80°C until analysis. AMH, inhibin A and estradiol concentrations were measured with enzyme-linked immunosorbent assays (Rat and Mouse AMH ELISA, AL-113, Ansh Labs; Equine/Canine/Rodent Inhibin A ELISA, AL-161, Ansh Labs; and Mouse/Rat Estradiol ELISA, ES180S-100, Calbiotech). Serum levels of FSH and LH were determined using the Luminex method (Oriental Yeast).

### Ovarian culture

Ovaries from mice at P4 were cultured on Transwell-COL membranes (3.0 µm pore size, Costar) for 6 days using the gas-liquid interphase method (Morohaku, 2019; Morohaku et al., 2016). The basal culture medium comprised α-minimum essential medium supplemented with 10% fetal bovine serum, 1.5-mM 2-*O*-α-D-glucopyranosyl-L-ascorbic acid (Tokyo Chemical Industry), and penicillin (10 U/ml)–streptomycin (10 µg/ml) (Sigma-Aldrich). Ovaries were treated with 2 μM IWP2 (Merck Millipore), 5 μM CHIR99021 (Sigma-Aldrich) or 100 μM 3BDO (Sigma-Aldrich), or with the corresponding concentration of DMSO as a vehicle control. Approximately half of the medium in each well was replaced with fresh medium every other day. The ovaries were maintained at 37°C under 5% CO_2_ and 95% air.

### Statistical analysis

All statistical analysis was performed using GraphPad Prism 8 or 9 software. Tests included the nonparametric Mann–Whitney matched-pairs test, unpaired multiple *t*-tests with the Holm-Sidak correction, two-way ANOVA with Sidak's post hoc test for multiple comparisons, and a chi-squared test for trend for the contingency table. A *P*-value of <0.05 was considered statistically significant.

## Supplementary Material

Supplementary information

Reviewer comments
