# Supplementary material for: WNT signaling in pre-granulosa cells is required for ovarian folliculogenesis and female fertility
Source: Development. 2021 Apr 29;148(9):dev198846. doi: 10.1242/dev.198846 (PMC8126407; doi:10.1242/dev.198846)
Supplement: Supplementary information [file develop-148-198846-s1.pdf]

Supplementary Information

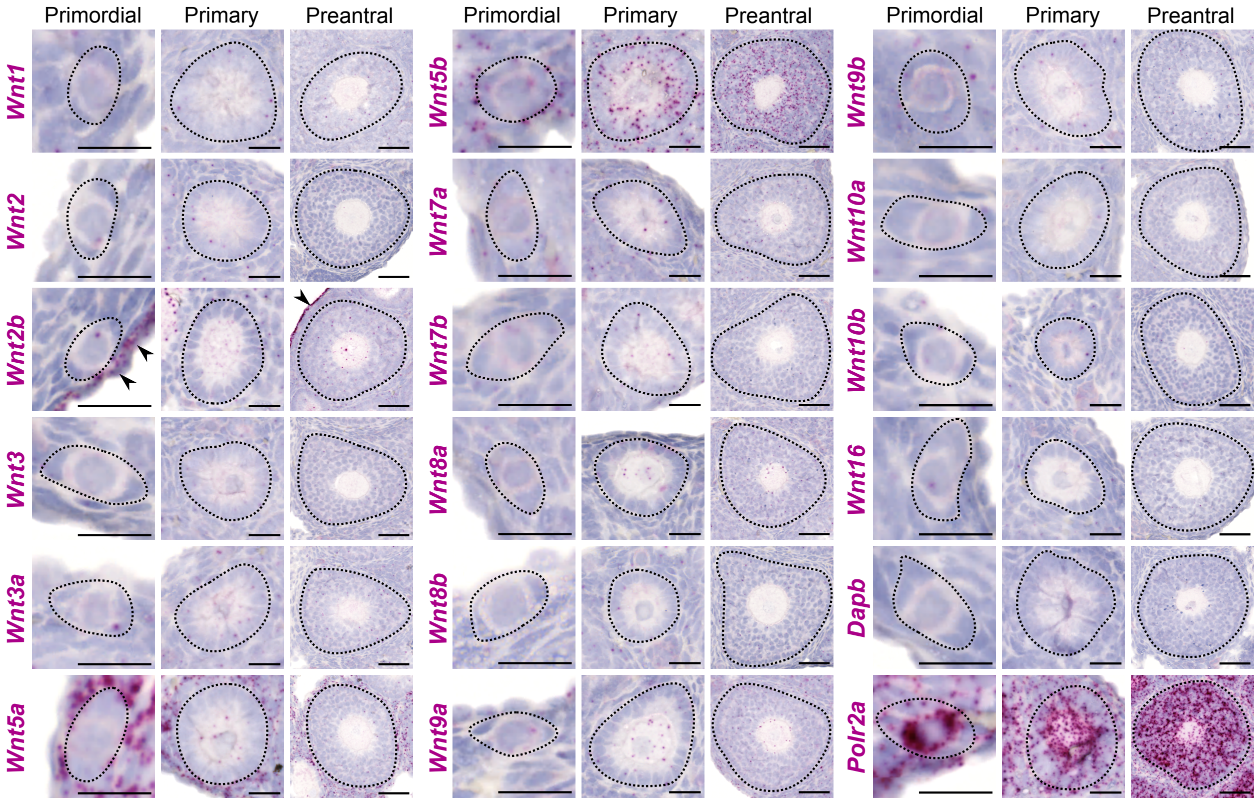

Figure S1. Expression pattern of Wnt ligands in mouse ovary.

In situ hybridization analysis of *Wnt1*, *Wnt2*, *Wnt2b*, *Wnt3*, *Wnt3a*, *Wnt5a*, *Wnt5b*, *Wnt7a*, *Wnt7b*, *Wnt8a*, *Wnt8b*, *Wnt9a*, *Wnt9b*, *Wnt10a*, *Wnt10b*, *Wnt16*, *Dapb* (negative control), and *Polr2a* (positive control) mRNAs (red) in ovaries of 3-week-old WT mice. Follicles were classified as primordial, primary, or preantral (dotted lines). Arrowheads indicate the ovarian epithelium. Scale bar, 50 µm (rightmost panels) or 20 µm (other panels).

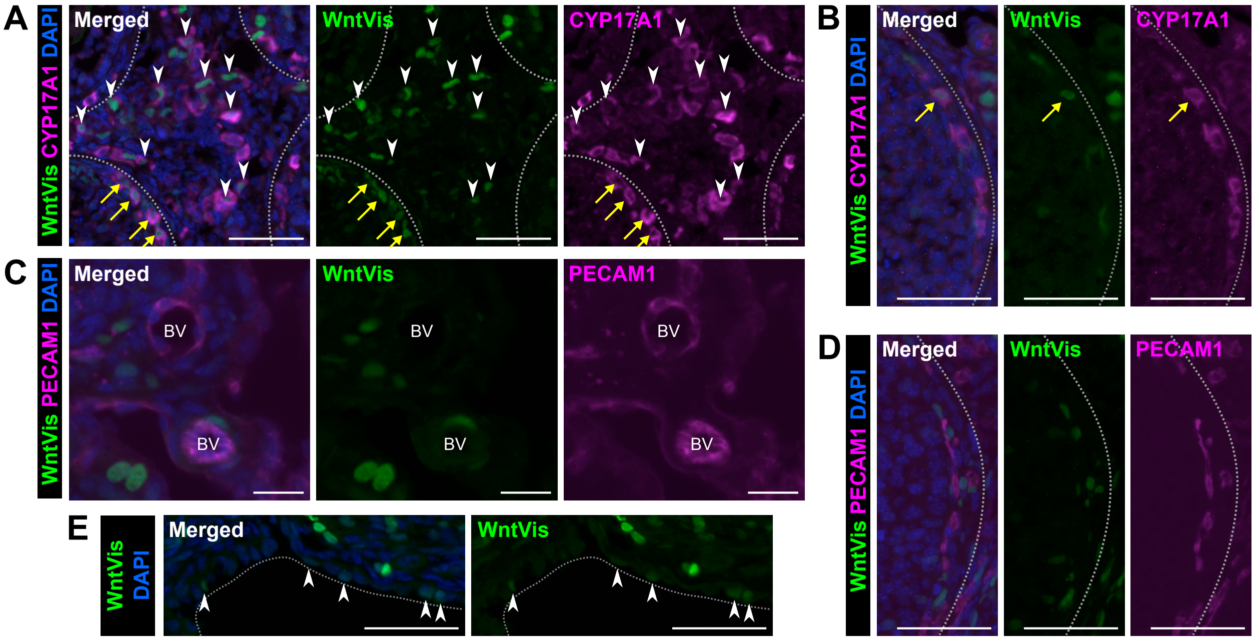

Figure S2. Interstitial cells, Theca cells, and ovarian epithelium receive Wnt signals.

(**A, B**) Immunofluorescence staining of WntVis (green) and CYP17A1 (magenta) in stromal (**A**) or Theca cell (**B**) regions of ovaries from 4-week-old *R26-WntVis* mice. Nuclei were counterstained with DAPI (blue). White arrowheads indicate interstitial cells which are double-positive for WntVis and CYP17A1. Yellow arrows indicate Theca cells which are double-positive for WntVis and CYP17A1. Gray dotted lines mark the boundaries of antral follicles. Scale bar, 50 µm.

(**C, D**) Immunofluorescence staining of WntVis (green) and PECAM1 (magenta) in blood vessels (**C**) or capillary vessels around follicles (**D**) of ovaries from 4-week-old *R26-WntVis* mice. Nuclei were counterstained with DAPI (blue). Gray dotted lines mark the boundaries of antral follicles. BV, blood vessels. Scale bar, 20 µm (**C**) or 50 µm (**D**).

(**E**) Immunofluorescence staining of WntVis (green) in ovarian epithelium of 4-week-old *R26-WntVis* mice. Nuclei were counterstained with DAPI (blue). The gray dotted lines mark the boundaries of ovaries. Scale bar, 50 µm. Arrowheads indicate WntVis-positive ovarian epithelium.

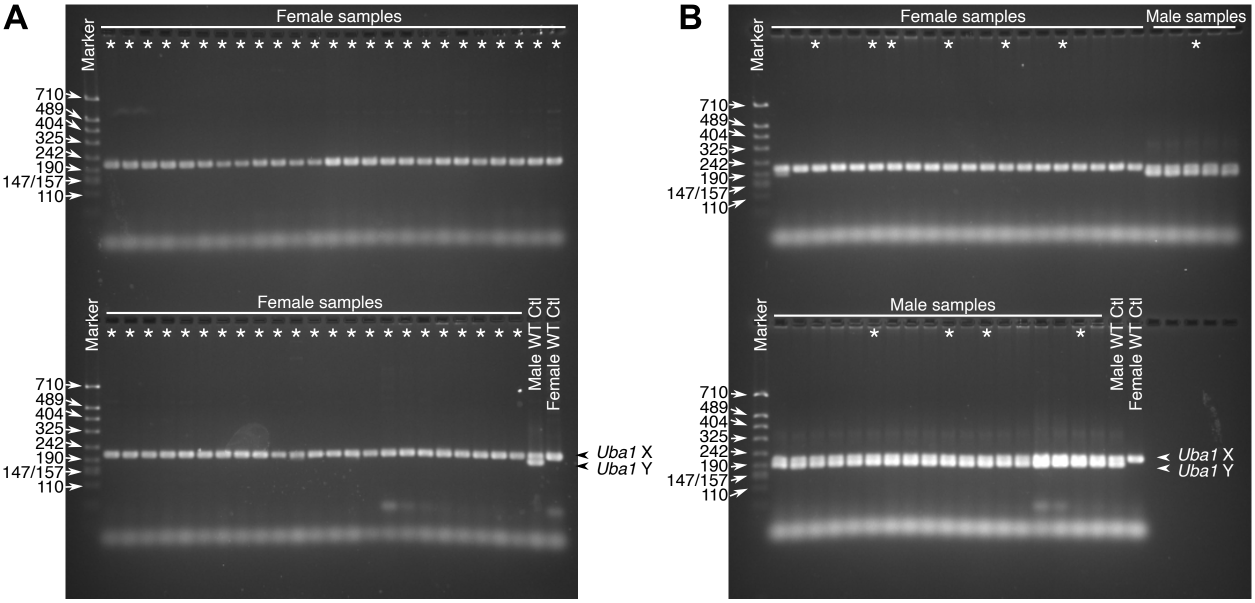

Figure S3. Sex genotyping of *Wls* cKO mice.

(**A, B**) Sex genotyping was performed on collected female *Wls* cKO mouse samples (**A**) or female/male mice obtained from a single in vitro fertilization (**B**). PCR-amplified products using primer sets for *Uba1* gene were analyzed by agarose gel electrophoresis with full images. Female product, single band of 217 bp; male samples, distinct bands of 198 and 217 bp. Each lane represents one independent sample. Asterisks indicate *Wls* cKO mice. Male or female wildtype (WT) mice were used as controls.

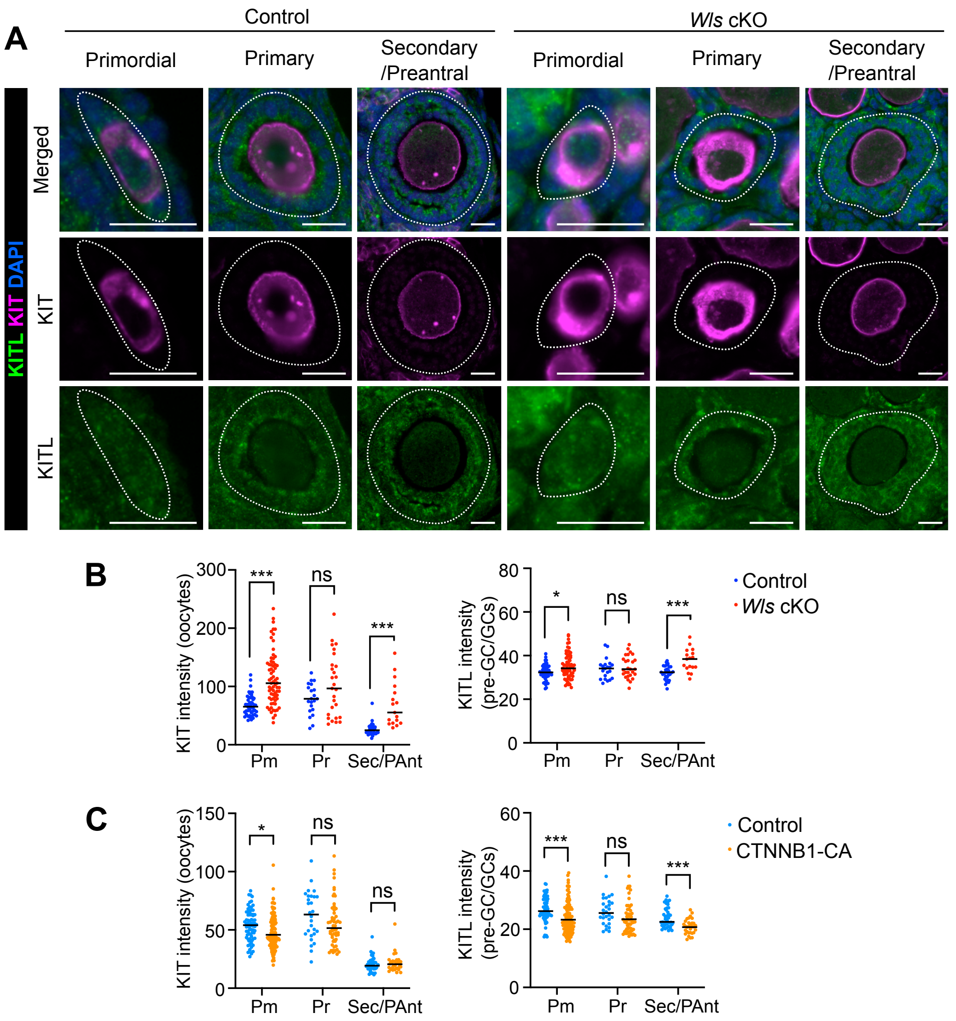

Figure S4. KIT and KITL expression in *Wls* cKO or CTNNB1-CA mice.

(**A**) Immunofluorescence staining of KITL (green) and KIT (magenta) in the ovaries of 3-week-old *Wls* cKO or littermate control mice. Nuclei were counterstained with DAPI (blue). Follicles are demarcated with white dotted lines. Scale bar, 20 µm.

(**B, C**) Fluorescence intensities of KIT in oocytes and KITL in pre-GC/GCs in the ovaries of *Wls* cKO (**B**), tamoxifen-treated CTNNB1-CA (**C**) or littermate control mice at 3 weeks of age. Follicles were morphologically classified (Pm, primordial; Pr, primary; Sec/PAnt, secondary/preantral). Horizontal lines represent the median. ns, not significant, $*P < 0.05$, $***P < 0.001$ (unpaired multiple *t* tests with Holm-Sidak correction). (n=110 follicles from five control mice; n=113 follicles from five *Wls* cKO mice (**B**). n=146 follicles from five control mice; n=203 follicles from five CTNNB1-CA mice (**C**).) The intensities from *Wls* cKO (**B**) and CTNNB1-CA (**C**) mice are not directly comparable because the data were obtained from different experiments.

Development • Supplementary information

	Primary					Secondary/Preantral				
	squamous	s/c	cuboidal	c/c	columnar	squamous	s/c	cuboidal	c/c	columnar
Control	6 (6.6%)	8 (8.8%)	12 (13.2%)	5 (5.5%)	8 (8.8%)	0 (0.0%)	1 (1.1%)	7 (7.7%)	0 (0.0%)	44 (48.4%)
Wls cKO	16 (26.2%)	12 (19.7%)	7 (11.5%)	1 (1.6%)	0 (0.0%)	0 (0.0%)	8 (13.1%)	17 (27.9%)	0 (0.0%)	0 (0.0%)

Table S1. Contingency table showing the phenotypic differences of developing follicles classified by GC morphology in 2-week-old *Wls* cKO (n = 61) and control (n = 91) mice.

	Primary					Secondary/Preantral				
	squamous	s/c	cuboidal	c/c	columnar	squamous	s/c	cuboidal	c/c	columnar
Control	2 (2.4%)	4 (4.9%)	15 (18.3%)	5 (6.1%)	4 (4.9%)	0 (0.0%)	0 (0.0%)	0 (0.0%)	1 (1.2%)	51 (62.2%)
PN-*Wls* cKO	42 (35.6%)	35 (29.7%)	12 (10.2%)	2 (1.69%)	1 (0.9%)	2 (1.69%)	11 (9.32%)	12 (10.2%)	1 (0.9%)	0 (0.0%)

Table S2. Contingency table showing the phenotypic differences of developing follicles classified by GC morphology in 3-week-old tamoxifen-treated PN-*Wls* cKO (n = 118) and control (n = 82) mice.
