# Supplementary material for: WNT signaling in pre-granulosa cells is required for ovarian folliculogenesis and female fertility
Source: Development. 2021 Apr 29;148(9):dev198846. doi: 10.1242/dev.198846 (PMC8126407; doi:10.1242/dev.198846)
Supplement: Reviewer comments [file develop-148-198846_review_history.pdf]

WNT signaling in pre-granulosa cells is required for ovarian folliculogenesis and female fertility

Okiko Habara, Catriona Y. Logan, Masami Kanai-Azuma, Roeland Nusse and Hinako M. Takase
DOI: 10.1242/dev.198846

Editor: Liz Robertson

Review timeline

Original submission:	19 November 2020
Editorial decision:	11 December 2020
First revision received:	3 March 2021
Editorial decision:	22 March 2021
Second revision received:	24 March 2021
Accepted:	26 March 2021

Original submission

First decision letter

MS ID#: DEVELOP/2020/198846

MS TITLE: Self-activation of Wnt signaling in pre-granulosa cells is required for ovarian folliculogenesis and female fertility

AUTHORS: Okiko Habara, Catriona Y Logan, Masami Kanai-Azuma, Roeland Nusse, and Hinako M Takase

I have now received all the referees' reports on the above manuscript, and have reached a decision. The referees' comments are appended below, or you can access them online: please go to BenchPress and click on the 'Manuscripts with Decisions' queue in the Author Area.

As you will see, the referees express considerable interest in your work, but have some significant criticisms and recommend a substantial revision of your manuscript before we can consider publication. If you are able to revise the manuscript along the lines suggested, which may involve further experiments, I will be happy receive a revised version of the manuscript. Your revised paper will be re-reviewed by one or more of the original referees, and acceptance of your manuscript will depend on your addressing satisfactorily the reviewers' major concerns. Please also note that Development will normally permit only one round of major revision.

We are aware that you may be experiencing disruption to the normal running of your lab that make experimental revisions challenging. If it would be helpful, we encourage you to contact us to discuss your revision in greater detail. Please send us a point-by-point response indicating where you are able to address concerns raised (either experimentally or by changes to the text) and where you will not be able to do so within the normal timeframe of a revision. We will then provide further guidance. Please also note that we are happy to extend revision timeframes as necessary.

Please attend to all of the reviewers' comments and ensure that you clearly highlight all changes made in the revised manuscript. Please avoid using 'Tracked changes' in Word files as these are lost in PDF conversion. I should be grateful if you would also provide a point-by-point response detailing

© 2021. Published by The Company of Biologists under the terms of the Creative Commons Attribution License (https://creativecommons.org/licenses/by/4.0/).

how you have dealt with the points raised by the reviewers in the 'Response to Reviewers' box. If you do not agree with any of their criticisms or suggestions please explain clearly why this is so.

Reviewer 1

Advance summary and potential significance to field

This manuscript provided by Habara et al. shows roles of WNT-signaling on primordial follicle activation (PFA). The authors first identified WNT ligands expressed in the primordial follicle by in situ hybridization and then investigated the function of the signaling. Based on the study using a number of genetically modified mice the authors found WNT-signaling had an autocrine effect on proliferation of pre-granulosa cells (pGCs) in a subset of primordial follicles, which triggers differentiation into granulosa cells (GCs). The authors confirmed these findings by in vitro culture system with Wnt-agonist or antagonist, which also provides an alternative tool for in vitro maturation of oocytes.

Comments for the author

This is an outstanding work that gains our understanding of mechanisms underlying PFA. Experiments in this paper are comprehensively and clearly done, therefore convincing their conclusion. There are several comments, some of which may be critical, below to be considered for further improvement of this paper. However, this reviewer overall supports publication of this paper in Development.

Comments
1. The authors should reconsider the abstract, since some sentences are peculiar. For example, it sounds repetitive: Inhibition of Wnt ligand secretion from pre-GCs/GCs led to female infertility due to impaired pre-GC differentiation, whereas constitutive stabilization of β-catenin induced thickening of the pre-GCs. Furthermore, a Wnt inhibitor suppressed pre-GC differentiation, while a Wnt activator rescued the Wls cKO phenotype in ovarian culture. Therefore, at least based on the abstract, “a two-step model of PFA” is irrational.
In addition, abbreviations, such as Wls and cKO, appear without clear definition.
2. L148, the author just mentioned “as confirmed by sex genotyping”. The author should exhibit the experimental data of the genotyping. Or alternatively, the author shoud mention the male:female ratio of the pups, as the authors deny the possibility (defect in sex determination) by administration of tamoxifen after birth.
3. L208, speculation for the reason of the comparable E2 level is poor. Is aromatase expressed in pGCs? Considering that 8 wks-old were used for this analysis, even if this production is independent of Wnt siganling, the level of E2 should be different. The author should consider another possibility such as involvement of extragonadal E2 production.
4. It is interesting and important that Foxoo3 was mostly in nuclei of Wls cKO oocytes as well as of Catnb-CA oocytes. This is puzzling. Can the author determine the level of expression of KITL (or Kitl) in these mice? Or alternatively, is there any feasible explanation of this?
5. It is very important to document Wnt-VIS negative follicles, as 50% of primordial follicles are negative. This affect the model shown in Figure 6.

Minor comments
1. Cite a reference for Sf1-Cre (L113).
2. Specify the project ID for the animal experiment.
3. Follow the regulation for nomenclature of gene and protein throughout the manuscript.
4. L708, Ddx4+ should be DDX4-positive in accordance with other words.
5. Consolidate the sequence of Figure 2 legend, since it is hardly readable.

Reviewer 2

Advance summary and potential significance to field

The manuscript by Habara et al., investigated the role of Wnt signaling in the process of pre-granulosa cells (pre-GCs) to granulosa cells (GCs) transition during primordial follicle activation.

© 2021. Published by The Company of Biologists under the terms of the Creative Commons Attribution License (https://creativecommons.org/licenses/by/4.0/).

The authors first used Sf1-cre;Wls-flox mice to knocked out Wntless in fetal ovarian somatic cells. In the mutant ovaries, pre-GCs failed to make the transition into GCs both morphologically and functionally, despite that enlarged growing oocytes were found in these follicles. The authors further used tamoxifen inducible creER mice (Wt1creERT2) to conditionally knockdown Wntless in postnatal ovarian somatic cells, and observed a similar defect in pre-GCs to GCs transition. The expression of beta-catenin (a downstream component of canonical Wnt singling pathway) driven by Wt1creERT2 resulted in primordial follicles that contained cuboidal GCs. Further more, by using in vitro ovary culture, the defect in pre-GCs to GCs transition in Wls mutant ovaries was rescued by a Wnt signaling activator. In summary, the results reported in the manuscript indicate a novel role of Wnt signaling in primordial follicle activation.

Comments for the author

In the manuscript, extensive studies have been done to demonstrate the novel role of Wnt signaling in the transition from pre-GCs to GCs during primordial follicle activation. The following concerns need to be addressed properly by the authors.

1. The authors used a Sf1-cre mouse line and a tamoxifen inducible Wt1creER line to drive Wntless knockout in ovarian somatic cells. However, the specificity and the knockout efficiency of these two cre lines were not addressed in the manuscript. It has been shown in previous studies that Sf1 is expressed in the progenitor cells that give rise to both pre-GCs and theca cells in fetal ovaries. This raises a concern over whether the defect in pre-GCs development are due to disrupted Wnt signaling in theca cells.
2. Based on the data from Wls cko mice in Fig 2B-E, the authors found that pre-GCs failed to make the transition to GCs despite oocyte activation. However, in fig1D and fig3A, developing follicles with cuboidal-shaped GCs were observed. It appears that in some follicles, despite the lack of Wnt singling, pre-GCs were able to make the transition to GCs and undergo proliferation. Thus, it is important to know what percentage of follicles showed the defect in pre-GC to GC transition. This should be addressed carefully in the phenotype observed in Wt1creER;Wls-flox mice as well. Line 307-308, this statement is not consistent with the results shown in fig1D and fig3A.
3. Many terms in the manuscript were not used properly, thus are misleading. For example, self-activation, oocyte reawakening, pre-GC differentiation, thickening of the preGC. There was no evidence in the manuscript showing that Wnt signaling is self-activated. It was not clear which biological processes the terms 'oocyte reawakening' and 'thickening of the preGC' refer to. The process of pre-GC to GC transition is not a differentiation process. Differentiation refers to a developmental process through which progenitor cells become the cells with different functions and molecular features.
4. 'Secondary follicle' refers to follicles with two layers of granulosa cells. Follicles with more than two layers of granulosa cells are referred to as preantral follicles. Please correct this.
5. Line 149, it is an overstatement. Whether cyst breakdown was effected or not was not shown in the manuscript.
6. Line 108, the authors stated that 'We did not observe intense expression of Wnt ligands in oocytes'. However, in fig S1, in the wnt2, wnt2b, wnt9a, wnt5b, wnt16, wnt11 probed ovarian sections, positive foci were observed in primary oocytes.
7. Line 306, in the 'two-step model' proposed by the authors, oocyte activation takes place after pre-GC to GC transition. However, this model is not consistent with the results in the manuscript. In the ovaries of Wls cko mice, oocytes were activated and grow in size despite that pre-GC remained flattened and failed to make the transition to GCs.
8. It is established that Wnt signaling is involved in ovarian development. The authors did not observe any defects in sex-differentiation and ovarian development in Wls cko mice. This difference in the results between the present study and previous studies should be discussed properly.
9. In WT follicle, FOXO3 is absent in the nuclei of developing oocytes. It is very interesting that in Wls cko ovaries, FOXO3 remained positive in the nucleus of the growing oocyte with multiple layers of GCs (fig 3I). This observation should be discussed properly.
10. In fig3A, it appears that FOXL2 was detected in the nuclei of oocytes in later stage follicles as well. Please confirm this result.
11. Line 267, Theca cells, T should be in lowercase.
12. Line 324, 'The activation of Wnt signaling …..expression pattern of Wnt4/6/11'. This sentence is confusing. Wnt4/6/11 are expressed in primordial, primary and preantral follicles. It was not clear

© 2021. Published by The Company of Biologists under the terms of the Creative Commons Attribution License (https://creativecommons.org/licenses/by/4.0/).

how the expression pattern of these ligands indicates that they are involved in primordial follicle activation?
13. Line 339-353, the authors discussed in vitro gametogenesis (a process that derives ESCs/iPSCs into oocytes). It was not clear how in vitro gametogenesis is relevant to the transition of pre-GCs to GCs.

First revision

Author response to reviewers' comments

We are grateful to reviewer 1 for the critical comments and useful suggestions that have helped us to improve our paper considerably. As indicated in the responses that follow, we have taken all these comments and suggestions into account in the revised version of our paper.

Comments by reviewer 1.

1. The authors should reconsider the abstract, since some sentences are peculiar. For example, it sounds repetitive: Inhibition of Wnt ligand secretion from pre-GCs/GCs led to female infertility due to impaired pre-GC differentiation, whereas constitutive stabilization of β-catenin induced thickening of the pre-GCs. Furthermore, a Wnt inhibitor suppressed pre-GC differentiation, while a Wnt activator rescued the Wls cKO phenotype in ovarian culture. Therefore, at least based on the abstract, "a two-step model of PFA" is irrational. In addition, abbreviations, such as Wls and cKO, appear without clear definition.

Response. In the revised manuscript, the abstract has been rewritten to eliminate the description of the results of in vitro experiments, since the data were similar to the results of in vivo experiments. We also reconsidered the 2-step model and changed it to a simple description. Correct definitions were added for terms of *Wntless* (*Wls*) and conditional knockout (cKO).

2. L148, the author just mentioned "as confirmed by sex genotyping". The author should exhibit the experimental data of the genotyping. Or alternatively, the author shoud mention the male:female ratio of the pups, as the authors deny the possibility (defect in sex determination) by administration of tamoxifen after birth.

Response. We have added the experimental data of sex genotyping as Fig. S3 and provided the protocol in the supplementary materials and methods section. We also described the data of the male/female ratio of *Sf1-Cre;Wls*$^{flox/del}$ (*Wls* cKO) mice and their siblings from the breeding pairs in lines 132–136. We confirmed there is no significant difference by the chi-square test.

3. L208, speculation for the reason of the comparable E2 level is poor. Is aromatase expressed in pGCs? Considering that 8 wks-old were used for this analysis, even if this production is independent of Wnt siganling, the level of E2 should be different. The author should consider another possibility such as involvement of extragonadal E2 production.

Response. A speculation of the cause was added on lines 232–239 to indicate that estrogen levels were not significantly reduced in *Wls* cKO mice. We speculate a compensatory effect, due to the fact that certain amounts of estrogen are detected in ovariectomized mice.

In addition to the probable causes described in the manuscript, there is another possible reason. Since estradiol is usually specifically elevated in Proestrus, urinal sampling was planned at the Proestrus stage. However, due to the influence of COVID19, it was not possible to enter the mouse room on a daily basis, and sampling was forced at a random estrous cycle. It is possible that the sampling conditions were not optimal. We added a statement to the Methods section that body fluids were sampled at random estrous cycles on line 628.

4. It is interesting and important that Foxo3 was mostly in nuclei of Wls cKO oocytes as

© 2021. Published by The Company of Biologists under the terms of the Creative Commons Attribution License (https://creativecommons.org/licenses/by/4.0/).

well as of Catnb-CA oocytes. This is puzzling. Can the author determine the level of expression of KITL (or Kitl) in these mice? Or alternatively, is there any feasible explanation of this?

Response. We performed immunostaining for KIT and KITL on ovarian samples of *Wls* cKO and CTNNB1-CA mice, and then measured the signal intensities. These results were included as Fig. S4. The description of *Wls* cKO data was added in lines 280–286, and the description of CTNNB1-CA data was added in lines 308–313. The conditions of immunostaining were described in the Supplementary Materials and Methods section.

Interestingly, KIT and KITL expression were elevated in *Wls* cKO mice and opposite phenotype was found in *Wt1*^{CreERT2};*Ctnnb1*$^{lox(ex3)/+}$ (CTNNB1-CA) mice. We currently do not yet understand how loss of WNT signaling might increase KIT and KITL. However, these data suggest that in addition to KIT signaling, unknown factors may act downstream of Wnt signaling to impact FOXO3 activity. In the future, measurement of KIT signaling activity by detecting phosphorylation of KIT or AKT, and transcriptomics on *Wls* cKO mice may help us further understand the signals that impact FOXO3 activity.

As reviewer 1 mentioned, primordial follicles of CTNNB1-CA mice show FOXO3 localization in the nuclei, similar to controls. We interpreted this to be due to the permissive role of WNT signaling and added the description to lines 306–308.

5. It is very important to document Wnt-VIS negative follicles, as 50% of primordial follicles are negative. This affect the model shown in Figure 6.

Response. Statements that we made were more ambiguous than intended, and we have adjusted to the text to be clearer on line 151–154. Although about 40% of pre-GCs (but not primordial follicles) were negative for WntVis, we consider most primordial follicles are receiving WNT signaling.

Minor comments by reviewer 1.

1. Cite a reference for Sf1-Cre (L113).
Response. We cited the reference for *SF1*-Cre and also the reference for *Wls* mouse in the same place (lines 124-125) to align the format.

2. Specify the project ID for the animal experiment.
Response. The approval number for the animal experiments was added on line 463.

3. Follow the regulation for nomenclature of gene and protein throughout the manuscript.
Response. We apologize for this error, and we have corrected it throughout the manuscript.

4. L708, Ddx4+ should be DDX4-positive in accordance with other words.
Response. We have corrected this mistake as suggested on line 900.

5. Consolidate the sequence of Figure 2 legend, since it is hardly readable.
Response. Fig. 2 legend has been rewritten in order to improve readability, line 896–924

We are grateful to reviewer 2 for the critical comments and useful suggestions that have helped us to improve our paper considerably. As indicated in the responses that follow, we have taken all these comments and suggestions into account in the revised version of our paper.

Comments by reviewer 2.

1. The authors used a Sf1-cre mouse line and a tamoxifen inducible Wt1creER line to drive Wntless knockout in ovarian somatic cells. However, the specificity and the knockout efficiency of these two cre lines were not addressed in the manuscript. It has been shown in previous studies that Sf1 is expressed in the progenitor cells that give rise to both pre-GCs and theca cells in fetal ovaries. This raises a concern over whether the defect in pre-GCs development are due to disrupted Wnt signaling in theca cells.

© 2021. Published by The Company of Biologists under the terms of the Creative Commons Attribution License (https://creativecommons.org/licenses/by/4.0/).

Response. We examined the efficiency of Cre recombination in FOXL2-positive GC for two strains, *SF1*-Cre and *WT1*^{CreERT2}, by crossing them with a reporter strain, *Ai9*. The efficiency of *SF1*-Cre is described in lines 128–131, and that of *WT1*^{CreERT2} is described in lines 205–207. All the data showed high Cre efficiency in GCs, over 97%. In Materials and Methods, we included the *Ai9* mouse strain on line 449, the protocol for immunostaining on lines 539–543, and the method for calculating Cre efficiency on lines 565–569.

We are aware of the concern that theca cell-derived Wnt ligand secretion is also suppressed in *Sf1-Cre;Wls*$^{flox/del}$ (*Wls* cKO) and *Wt1*^{CreERT2};*Wls*$^{flox/del}$ (PN-*Wls* cKO) mice. Using 4-week-old *SF1*-Cre;*Ai9* mice, we calculated the Cre efficiency in CYP17A1-positive theca cells as 97.8% ± 1.3%. This is high, but unfortunately, we were unable to calculate it in mice younger than 4 weeks of age, because theca cell markers could not be immunostained while retaining Ai9 fluorescence. For this reason, we have not included this data in the manuscript.

The expression of *Wnt4*, *Wnt5a*, and *Wnt5b* can be detected in theca and stromal cell regions. We feel that the importance of theca cell derived WNTs should be investigated in follow-up studies using different genetically modified mice.

2. Based on the data from Wls cko mice in Fig 2B-E, the authors found that pre-GCs failed to make the transition to GCs despite oocyte activation. However, in fig1D and fig3A, developing follicles with cuboidal-shaped GCs were observed. It appears that in some follicles, despite the lack of Wnt singling, pre-GCs were able to make the transition to GCs and undergo proliferation. Thus, it is important to know what percentage of follicles showed the defect in pre-GC to GC transition. This should be addressed carefully in the phenotype observed in Wt1creER;Wls-flox mice as well. Line 307- 308, this statement is not consistent with the results shown in fig1D and fig3A.

Response. We re-analyzed the growing follicles of *Wls* cKO and PN-*Wls* cKO mice to answer this useful comment, since we did not classify the morphology of GCs as cuboidal or columnar in the previous version. The data can be found in Table S1 and Table S2. The results of *Wls* cKO are described in lines 174–180, and the results of PN-*Wls* cKO in lines 210–211. As pointed out, we found that even when WNT signaling is suppressed, some follicles have cuboidal GCs, and we clarified this in line 180–182. The method for counting is described as "Morphometric analyses of growing follicles" in Supplementary Materials and Methods. We have added the word “columnar” to the text in line 63 to be consistent. The statement on lines 360–362 has been weakened to be consistent with the new data.

3. Many terms in the manuscript were not used properly, thus are misleading. For example, self- activation, oocyte reawakening, pre-GC differentiation, thickening of the preGC. There was no evidence in the manuscript showing that Wnt signaling is self-activated. It was not clear which biological processes the terms 'oocyte reawakening' and 'thickening of the preGC' refer to. The process of pre-GC to GC transition is not a differentiation process. Differentiation refers to a developmental process through which progenitor cells become the cells with different functions and molecular features.

Response. We apologize for the inappropriate wording. We have either removed the word “self-activation” or replaced it with the word “autocrine”, including the title and line 358. Throughout the manuscript, instead of saying that pre-GC "differentiates" into GC, we wrote "transitions". The abstract was extensively rewritten, but the description of "thickening of the pre-GCs" has been replaced with "morphological change of pre-GCs from a squamous into a cuboidal form" on lines 38-39.

4. 'Secondary follicle' refers to follicles with two layers of granulosa cells. Follicles with more than two layers of granulosa cells are referred to as preantral follicles. Please correct this.

Response. We apologize for this error. The related mistakes throughout the manuscript and the figures have been corrected as pointed out.

5. Line 149, it is an overstatement. Whether cyst breakdown was effected or not was

© 2021. Published by The Company of Biologists under the terms of the Creative Commons Attribution License (https://creativecommons.org/licenses/by/4.0/).

not shown in the manuscript.

Response. We deleted the statement of cyst breakdown from line 166.

6. Line 108, the authors stated that 'We did not observe intense expression of Wnt ligands in oocytes'. However, in fig S1, in the wnt2, wnt2b, wnt9a, wnt5b, wnt16, wnt11 probed ovarian sections, positive foci were observed in primary oocytes.

Response. Thank you for valid comment. To accurately describe the expression of the *Wnt* ligands, we added a sentence in lines 116–117.

7. Line 306, in the 'two-step model' proposed by the authors, oocyte activation takes place after pre-GC to GC transition. However, this model is not consistent with the results in the manuscript. In the ovaries of Wls cko mice, oocytes were activated and grow in size despite that pre-GC remained flattened and failed to make the transition to GCs.

Response. Thank you for pointing out this important point. We meant that the oocyte activation does actually occur in *Wls* cKO mice with increasing in size, but that process is not complete as the nuclear- cytoplasmic shuttling of FOXO3 is inhibited. However, we agree that our 2-step model was unclear in the original manuscript. We have simplified the explanation in the abstract (lines 39–41) and the discussion (lines 358–360), as well as Fig. 6.

8. It is established that Wnt signaling is involved in ovarian development. The authors did not observe any defects in sex-differentiation and ovarian development in Wls cko mice. This difference in the results between the present study and previous studies should be discussed properly.

Response. We created a new paragraph in lines 396–417 to discuss this issue. To support our hypothesis that sex determination is not affected in *Wls* cKO mice, we have added the experimental data of sex genotyping as Fig. S3. The male/female ratio of *Wls* cKO mice and their siblings were described in lines 132–136. We confirmed there is no significant difference by the Chi-square test.

9. In WT follicle, FOXO3 is absent in the nuclei of developing oocytes. It is very interesting that in Wls cko ovaries, FOXO3 remained positive in the nucleus of the growing oocyte with multiple layers of GCs (fig 3I). This observation should be discussed properly.

Response. We confirmed that FOXO3 does remain in the nucleus of the oocyte in secondary follicles from *Wls* cKO mice mice. To clarify this point, we added a sentence to lines 267–269. We also performed immunostaining for FOXO3 again and replaced images in Fig. 3I. In *Wls* cKO mice, growing follicles are rare and poor in morphology with some background on GCs. However, the nearby oocytes of primordial follicles are clearly stained, thus we believe that the signal in the oocyte of growing follicles is FOXO3.

To further investigate the extent of oocyte activation in *Wls* cKO mice, phosphorylation of ribosomal protein S6 (RPS6), was detected by immunostaining and its intensity was measured. The results are shown in Fig. 3K-3L and described in lines 268–276. Together with the FOXO3 data, our new data provides strong evidence that the WNT signaling-mediated transition from pre-GC to GC is important for oocyte activation.

10. In fig3A, it appears that FOXL2 was detected in the nuclei of oocytes in later stage follicles as well. Please confirm this result.

Response. We have confirmed that the immunostaining in the original figure used the correct FOXL2 antibody to stain the target GCs. However, we apologize for the background signal that was visible in the nuclei of the oocytes, as pointed out. By performing the immunostaining again with a different combination of secondary antibody dyes, we were able to obtain a clearer picture. Figure images in Fig. 3A have been replaced.

11. Line 267, Theca cells, T should be in lowercase.

© 2021. Published by The Company of Biologists under the terms of the Creative Commons Attribution License (https://creativecommons.org/licenses/by/4.0/).

Response. We apologize for this error, and we have corrected it on lines 148 and 321.

12. Line 324, 'The activation of Wnt signaling …..expression pattern of Wnt4/6/11'. This sentence is confusing. Wnt4/6/11 are expressed in primordial, primary and preantral follicles. It was not clear how the expression pattern of these ligands indicates that they are involved in primordial follicle activation?

Response. To make this confusing wording clearer, we added an additional explanation in lines 382-383. *Wnt4/6/11* is strongly expressed in the pre-GC/GCs of primordial and primary follicles, but expression is reduced as the follicle grows into secondary and preantral follicles.

13. Line 339-353, the authors discussed in vitro gametogenesis (a process that derives ESCs/iPSCs into oocytes). It was not clear how in vitro gametogenesis is relevant to the transition of pre-GCs to GCs.

Response. We hypothesized that if the somatic cells used in vitro gametogenesis were activated more efficiently, more oocytes would grow and be functional, but we agree it was overstated. The content was reconsidered and the discussion on in vitro gametogenesis was removed and the potential of WNT administration was discussed mainly for in vitro activation (lines 419-445).

Second decision letter

MS ID#: DEVELOP/2020/198846

MS TITLE: WNT signaling in pre-granulosa cells is required for ovarian folliculogenesis and female fertility

AUTHORS: Okiko Habara, Catriona Y Logan, Masami Kanai-Azuma, Roeland Nusse, and Hinako M Takase

I have now received all the referees reports on the above manuscript, and have reached a decision. The referees' comments are appended below, or you can access them online: please go to BenchPress and click on the 'Manuscripts with Decisions' queue in the Author Area.

The overall evaluation is very positive and we would like to publish a revised manuscript in Development. However as you will see Reviewer 2 suggests 2 extremely minor changes. I am returning the manuscript to give you the opportunity to make this final change which hopefully will only take a matter of minutes. As soon as you submit the final version I will accept the paper.

We are aware that you may currently be unable to access the lab to undertake experimental revisions. If it would be helpful, we encourage you to contact us to discuss your revision in greater detail. Please send us a point-by-point response indicating where you are able to address concerns raised (either experimentally or by changes to the text) and where you will not be able to do so within the normal timeframe of a revision. We will then provide further guidance. Please also note that we are happy to extend revision timeframes as necessary.

Reviewer 1

Advance summary and potential significance to field

In this study, mainly based on genetic analyses, the authors found WNT-signaling had an autocrine effect on proliferation of pre-granulosa cells (pGCs) in a subset of primordial follicles, which triggers differentiation into granulosa cells (GCs). The authors confirmed these findings by in vitro culture system with Wnt-agonist or antagonist, which also provides an alternative tool for in vitro

© 2021. Published by The Company of Biologists under the terms of the Creative Commons Attribution License (https://creativecommons.org/licenses/by/4.0/).

maturation of oocytes. This study provides a significant insight into a fundamental question how follicular activation occurs.

Comments for the author

In the revised manuscript, the authors appropriately address the comments with additional experiments.
Especially an increased KIT/KITL in WlscKO is striking and provides a new question to be addressed in future.
This reviews recommends publication of this manuscript in Development.

Reviewer 2

Advance summary and potential significance to field

The manuscript by Habara et al., investigated the role of Wnt signaling in the process of pre-granulosa cells (pre-GCs) to granulosa cells (GCs) transition during primordial follicle activation. The authors first used Sf1-cre;Wls-flox mice to knocked out Wntless in fetal ovarian somatic cells. In the mutant ovaries, pre-GCs failed to make the transition into GCs both morphologically and functionally, despite that enlarged growing oocytes were found in these follicles. The authors further used tamoxifen inducible creER mice (Wt1creERT2) to conditionally knockdown Wntless in postnatal ovarian somatic cells, and observed a similar defect in pre-GCs to GCs transition. The expression of beta-catenin (a downstream component of canonical Wnt singling pathway) driven by Wt1creERT2 resulted in primordial follicles that contained cuboidal GCs. Further more, by using in vitro ovary culture, the defect in pre-GCs to GCs transition in Wls mutant ovaries was rescued by a Wnt signaling activator. In summary, the results reported in the manuscript indicate a novel role of Wnt signaling in primordial follicle activation.

Comments for the author

1. Line 159. Maturation often refers to the final stage of folliculogenesis, I suggest replace the word 'maturation' with 'development'.
2. Regarding to the response to question 1, the authors' response addressed the concern sufficiently. Since the phenotype reported in the manuscript was from Sf1-cre mice, the potential role of Wnt signaling from theca cells in pregranulosa cell development and primordial follicle activation should be discussed in the manuscript.

Second revision

Author response to reviewers' comments

We are grateful to the reviewers for their precious time in reviewing our paper and providing valuable comments. We have taken all these comments into account in the revised version of our paper. Below we provide the point-by-point responses.

Reviewer 1 Comments.
In the revised manuscript, the authors appropriately address the comments with additional experiments.
Response. We highly appreciate the positive feedback from the reviewer.

Reviewer 2 Comment 1.
Maturation often refers to the final stage of folliculogenesis, I suggest replace the word 'maturation' with 'development'.
Response. We revised the manuscript accordingly.

© 2021. Published by The Company of Biologists under the terms of the Creative Commons Attribution License (https://creativecommons.org/licenses/by/4.0/).

Reviewer 2 Comment 2.
Regarding to the response to question 1, the authors' response addressed the concern sufficiently. Since the phenotype reported in the manuscript was from Sf1-cre mice, the potential role of Wnt signaling from theca cells in pregranulosa cell development and primordial follicle activation should be discussed in the manuscript.
Response. We added a sentence on lines 417–420 to mention a possible role of WNTs secreted from theca cells that can be addressed in future studies. On line 127, we clarify that WNT secretion is inhibited not only in granulosa cells but also theca cells.

Third decision letter

MS ID#: DEVELOP/2020/198846

MS TITLE: WNT signaling in pre-granulosa cells is required for ovarian folliculogenesis and female fertility

AUTHORS: Okiko Habara, Catriona Y Logan, Masami Kanai-Azuma, Roeland Nusse, and Hinako M Takase
ARTICLE TYPE: Research Article

I am happy to tell you that your manuscript has been accepted for publication in Development, pending our standard ethics checks.

© 2021. Published by The Company of Biologists under the terms of the Creative Commons Attribution License (https://creativecommons.org/licenses/by/4.0/).
